# A brief intervention for weight control based on habit-formation theory delivered through primary care: results from a randomised controlled trial

**DOI:** 10.1038/ijo.2016.206

**Published:** 2016-11-21

**Authors:** R J Beeken, B Leurent, V Vickerstaff, R Wilson, H Croker, S Morris, R Z Omar, I Nazareth, J Wardle

**Affiliations:** 1 Department of Epidemiology and Public Health, University College London, London, UK; 2 Department of Primary Care and Population Health, University College London, London, UK; 3 Department of Statistical Science, University College London, London, UK

**Keywords:** Obesity, Weight management, Randomized controlled trials, Health care economics

## Abstract

**Background:**

Primary care is the 'first port of call' for weight control advice, creating a need for simple, effective interventions that can be delivered without specialist skills. Ten Top Tips (10TT) is a leaflet based on habit-formation theory that could fill this gap. The aim of the current study was to test the hypothesis that 10TT can achieve significantly greater weight loss over 3 months than ‘usual care’.

**Methods:**

A two-arm, individually randomised, controlled trial in primary care. Adults with obesity were identified from 14 primary care providers across England. Patients were randomised to either 10TT or 'usual care' and followed up at 3, 6, 12, 18 and 24 months. The primary outcome was weight loss at 3 months, assessed by a health professional blinded to group allocation. Difference between arms was assessed using a mixed-effect linear model taking into account the health professionals delivering 10TT, and adjusted for baseline weight. Secondary outcomes included body mass index, waist circumference, the number achieving a 5% weight reduction, clinical markers for potential comorbidities, weight loss over 24 months and basic costs.

**Results:**

Five-hundred and thirty-seven participants were randomised to 10TT (*n*=267) or to ‘usual care' (*n*=270). Data were available for 389 (72%) participants at 3 months and for 312 (58%) at 24 months. Participants receiving 10TT lost significantly more weight over 3 months than those receiving usual care (mean difference =−0.87kg; 95% confidence interval: −1.47 to −0.27; *P*=0.004). At 24 months, the 10TT group had maintained their weight loss, but the ‘usual care’ group had lost a similar amount. The basic cost of 10TT was low, that is, around £23 ($32) per participant.

**Conclusions:**

The 10TT leaflet delivered through primary care is effective in the short-term and a low-cost option over the longer term. It is the first habit-based intervention to be used in a health service setting and offers a low-intensity alternative to ‘usual care’.

**Supplementary information:**

The online version of this article (doi:10.1038/ijo.2016.206) contains supplementary material, which is available to authorized users.

## Introduction

Following marked rises in obesity rates over the past three decades, global prevalence of obesity is now around 10%, and in the United Kingdom more than a quarter of adults are obese.^1^ The health risks associated with excess weight,^2^ and the costs to health services,^3^ mean there is an urgent need to find effective interventions that can be delivered on a wide scale to both prevent and treat obesity.

Health professionals within primary care are well placed to deliver first-line interventions. The latest guidance in both the United States^4^ and the United Kingdom^5^ makes direct recommendations for obesity to be addressed within primary care, and suggests that just 3% weight loss may improve long-term health outcomes. However, few health professionals routinely give weight loss advice,^6, 7, 8, 9, 10, 11^ with insufficient time, lack of knowledge, training or confidence and inadequate materials reported to be major barriers.^12, 13, 14, 15, 16, 17, 18^

Referrals to commercial weight management programme have been shown to be effective in primary care populations.^19, 20^ However, they typically require high commitment; for example, attendance at group sessions on a weekly basis for at least 3 months. Patients with less strong motivation may be reluctant to take up this kind of referral, and it may be practically difficult for some. Some patients also have a preference for weight management delivered through primary care.^21^ Brief interventions that enable health professionals to deliver effective advice in primary care could provide an important tool.

Habit-formation theory offers a unique perspective from which to derive a new approach to weight management. Habits are automatically triggered actions, learned through repetition of the action in a consistent context. Interventions targeting automatic actions have application where patients’ level of engagement or motivation means that more traditional behavioural approaches with inherently high demand for commitment achieve limited adherence.^22, 23^ Habit-based advice has been identified as particularly useful in primary care, as it is less time consuming to explain and easier for patients to implement than traditional behaviour change strategies.^22^ Recent studies have demonstrated the potential for habit-based interventions to encourage positive health behaviours.^24, 25^

We developed ‘Ten Top Tips’ (10TT), a self-guided leaflet for weight management focusing on making simple diet and exercise behaviours habitual. A small randomised trial (*n*=104) in a volunteer population found that 10TT produced significantly greater weight loss than a no-treatment control condition after 8 weeks (10TT: −2.0 kg; control: −0.4 kg), and weight loss continued over the longer term, reaching −3.6 kg in completers at 32 weeks follow-up.^26^ These findings made the case for an evaluation of the intervention in a full-size, randomised trial in the primary care clinical population.

### Primary research objective

We tested the hypothesis that 10TT offered to obese primary care patients would achieve a significantly greater loss in body weight over 3 months than ‘usual care’.

### Secondary research objectives

In our protocol,^27^ we described a number of secondary objectives. This paper focuses on differences in the main secondary outcomes at 3 months and over the 24-month trial period, namely, body mass index (BMI), waist circumference and the percentage of people achieving a 5% reduction in weight. We also explore differences in clinical markers for potential comorbidities (blood pressure, total cholesterol/low-density lipoprotein (LDL) and blood glucose) and changes in automaticity for the target behaviours at 3 months. Whether weight loss is maintained over 24 months (including measurements of weight loss at 6, 12 and 18 months) and the basic cost of 10TT are also examined.

## Materials and methods

### Design

The protocol for the study has been published.^27^ The trial was a multicentre, parallel, two-arm, individually randomised (1:1 allocation ratio), controlled trial in adults with obesity in primary care in England, testing the superiority of 10TT over usual care at 3 months, and including additional follow-ups at 6, 12, 18 and 24 months.

### Participants

Patients with obesity (BMI ⩾30 kg m^−^^2^) were identified from electronic primary care physician (PCP) records in 14 PCPs. A random sample of 500 patients with obesity from each PCP was selected to receive an invitation letter with an enclosed information sheet. PCPs stopped sending out invitations once their recruitment target had been met or the recruitment period came to an end. Recruitment took place from August 2010 to October 2011.

We restricted the study to adults (age ⩾18 years) who were able to consent for themselves. We targeted those with obesity (BMI ⩾30 kg m^−^^2^) and excluded anyone who was (i) unable to provide informed consent due to mental incapacity or active psychotic illness, (ii) pregnant or (iii) terminally ill.

### Setting

PCPs across England (*n*=14) were recruited through the MRC General Practice Research Framework. The majority were located in Southern England (*n*=9), with three in the Midlands and two in the North. The number of adult patients registered at each PCP ranged from 8424 to 22 466, and recorded rates of obesity on PCP databases ranged from 8.4 to 23.4%. Eight of the PCPs were in rural settings, and six were in urban settings. Ten PCPs were located within the two most socioeconomic deprived quintiles in England and the rest were in the second and third quintiles of deprivation.^28^

### Interventions

#### 10TT

We developed a simple, self-guided, leaflet-based intervention, 10TT. The intervention focused explicitly on the recommendations of habit-formation theory; thus, negative energy balance behaviours are listed alongside advice on repetition and context stability in the leaflet. Health professionals (nurses or health-care assistants) in each centre attended a training session, and were provided with a script to enable them to deliver the intervention in a standardised way. During the training, the importance of restricting the use of any aspect of 10TT to only those patients randomised to the 10TT group was emphasised to try and minimise contamination.

Immediately after randomisation, patients received the 10TT leaflet, together with a simple logbook for self-monitoring of target behaviours and weight during the 3-month habit acquisition phase, and a wallet sized card with guidance on food labels. A single 30 min session within the baseline appointment was allocated to take patients through the leaflet using a flip chart and defined script in line with previous recommendations for discussing habit formation in primary care.^22^ At 3 months, patients were mailed a second copy of the 10TT leaflet and were told they could request additional copies of the logbook. Quality checks involving site visits to observe the delivery of the intervention were carried out to ensure compliance. The leaflet, flip chart and script are all available as Supplementary Material to this article.

#### Usual care

Patients randomised to ‘usual care’ were referred to each PCPs usual care treatment, which they received either within their PCP at subsequent appointments (for example, from a dietitian) or from an external provider (for example, Weight Watchers). At 3 months, we recorded the strategies used by each PCP. At each follow-up, participants in both groups were also asked to report any additional weight loss programmes they had followed over the previous 3-month period.

### Outcome measures

#### Demographics

Demographic data (gender, date of birth, ethnicity and educational qualifications) were collected at baseline. Postcode of patient residence was recorded for linkage with the Index of Multiple Deprivation (IMD), obtained via the UK Data Service website.^28^ Based on the National Statistics Postcode Directory 2010, Indices of Deprivation 2007 for each participant’s postcode was matched to the corresponding LSOA (Lower layer Super Output Area) and IMD rank.

#### Primary outcome

The primary outcome of the trial was the change in measured weight (kg) between baseline and 3 months, measured using TANITA scales supplied to PCPs for use in this study.

#### Secondary outcomes

PCP equipment was used to measure height (cm) at baseline, so that BMI could be calculated using the standard formula (weight (kg/height (m)^2^). Waist circumference was measured using the point midway between the iliac crest (top of the hip bone) and the lower rib. Blood pressure and blood cholesterol/LDL/glucose levels were assessed at baseline and 3 months times according to standard PCP procedures. Automaticity of the target behaviours at baseline and at the end of the active intervention period was measured using the automaticity item from the Self-Report Habit Index^29^ for each of the target behaviours. An overall automaticity score was calculated by summing the individual scores for the target behaviours. Body weight and waist were measured again at 6, 12, 18 and 24 months. The basic cost of 10TT per participant and the cost of usual care were calculated at 24 months.

### Sample size and assumptions

We powered the study to detect a mean difference in weight change of 1.0 kg at 3 months, with a standard deviation of 2.5. This was based on the exploratory trial of 10TT,^26^ which showed a difference of weight change between baseline and 8 weeks of 1.41 kg (s.d.=1.9) between the intervention and control groups. We based our calculation on more conservative figures to take into account the likely increase in heterogeneity within the primary care population. Based on an average cluster size of 13 evaluable participants (those completing 3 months follow-up), and a therapist (health professional delivering the intervention) intraclass correlation coefficient of 0.05^(ref. 30)^ in the intervention arm, a total of 364 evaluable patients at 3 months would provide 92% power to detect such a difference with a two-sample test and a significance level of 5%.^31^ This ensured that the power would stay above 90% even after accounting for loss of power because of potential imbalance in cluster size between PCPs.^32^ Allowing for 30% attrition (26% observed in the pilot study at 8 weeks), 260 participants needed to be recruited in each arm, or 520 in total.

### Randomisation

A central telephone-based randomisation service (Health Service Research Unit at Aberdeen) was used to randomise at the level of the patient, ensuring allocation concealment. Randomisation took place after the patient had provided informed consent and baseline data, at which point the health professional carrying out the baseline assessment telephoned the randomisation service. They then either took the patient through the 10TT leaflet or referred them to the PCP’s usual care, based on allocation. A computer-generated list of random permuted blocks of size 2–4 was used. Randomisation was stratified by PCP to ensure socioeconomic balance between groups.

### Blinding

All measurements at 3 months were with a health professional blind to group allocation. All subsequent follow-ups were unblinded. Unblinding of the data and analysis was initiated after the last patient had completed 3 months of follow-up, all relevant data had been entered, data checking had been performed and the analysis plan was finalised and approved. Analysis programmes were prepared as much as possible before unblinding.

### Statistical analysis

A detailed analysis plan was written and published before initiation of the analysis.^33^ All analyses were according to randomisation arm, independently of whether or not patients received the allocated intervention (intention-to-treat). Statistical analysis was performed using STATA software (version 12/13, StataCorp LP, College Station, TX, USA). A window of plus or minus 6 weeks was allowed for observations of all outcomes.

#### Primary analysis

The difference in weight change between arms was estimated, adjusting for baseline weight. To take into account clustering by health professional delivering the 10TT, a random effect by health professional in the intervention group was added, and residuals were allowed to vary by trial arm. The normality assumptions of the residuals were satisfied. This corresponds to a heteroscedastic model for a partially nested design.^31, 34^ The model was also adjusted for the randomisation stratification variable (PCP) using a random effect by PCP.^35^ The primary analysis was based on observed outcome values (complete case), and performed by two statisticians separately to ensure its accuracy.

#### Secondary outcomes

Logistic or linear regression (as appropriate) was used to estimate differences between arms in secondary outcomes at 3 months, adjusted for baseline weight and baseline value of the outcome. As for the primary outcome, a random effect by PCP, and by health professional in the intervention group, was added to take account of the possible clustering variations.

To explore changes in weight over 2 years, we used a mixed-effect model, using all patient outcome data over 24 months, while taking into account the correlations between measurements from the same patient, and including random effects to take account of clustering by PCP. The model included baseline weight and a group by time interaction. A similar repeated-measure model was fitted over 24 months for the other secondary outcomes: 5% change in weight, change in BMI and change in weight circumference adjusted for the corresponding baseline measurement, baseline weight and a group × time interaction.

#### Sensitivity to missing outcome data

We performed sensitivity analyses under various assumptions for the missing primary outcomes. First, we fitted the primary analysis model adding the main predictors of missingness as covariates, then performed a ‘Baseline Observation Carried Forward’ analysis (replacing missing weight at 3 months by baseline weight). We also used multiple imputation by stratifying the imputation model by study arm, and included the outcome of interest (weight change), sociodemographics and anthropometrics data at baseline, and any other variables related to missingness or weight change. A set of 100 imputations were performed. Further sensitivity analyses considered the possibility of the data being Missing Not At Random (MNAR; the chances of being missing is dependent on the weight change itself) mechanisms. MNAR analyses were performed with the STATA user-written command rctmiss,^36^ using a pattern-mixture approach, and adjusted for baseline weight and PCP.

We also performed sensitivity analyses for the missing weight data over the 24-month period adjusting for predictors of missingness and using multiple imputations (*n*=100). The imputation was performed by study arm and included the weight from previous and subsequent time points along with sociodemographic and anthropometric data at baseline.

#### Basic costs

We calculated the cost of 10TT per participant based on the cost of materials and nurse time.^37^ Costs were calculated in 2014 UK£ and converted into 2014 US$ using GDP purchasing power parities.

## Results

**Figure 1:**
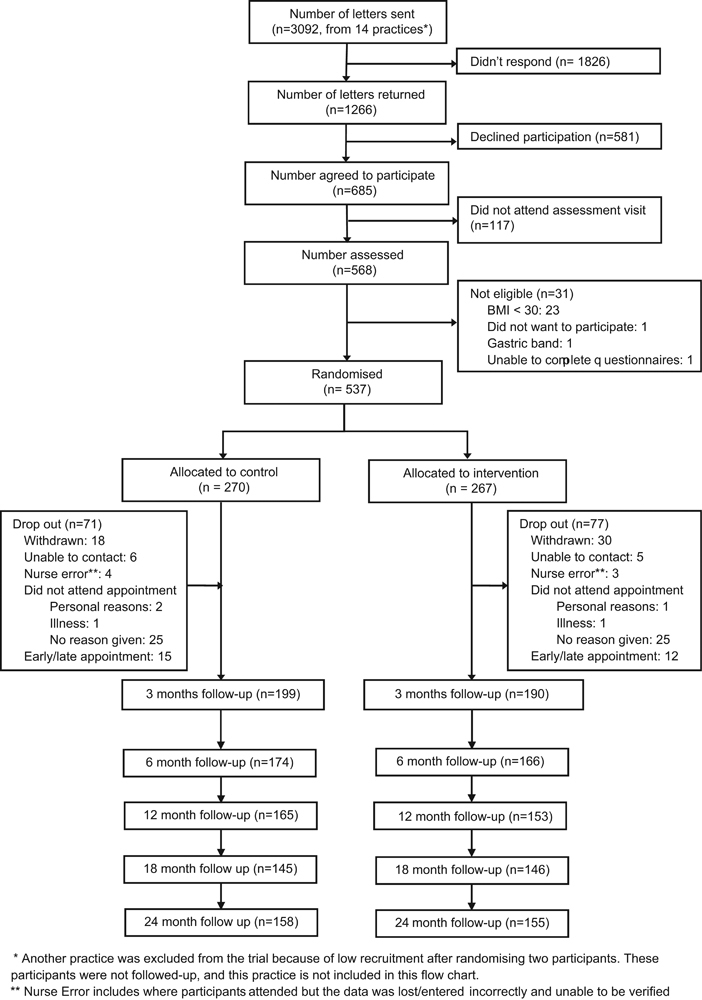
10 Top Tips flowchart.

Of the 3092 people invited, 537 (17.4%) consented to take part (Figure 1). The median number of patients recruited per PCP was 38 (range 21–51). Table 1 shows the baseline characteristics of the patients, which did not appear to differ by arm.

**Table 1:** Baseline characteristics

	*Usual care (*n=*270)*^a^		*10TT (*n=*267)*^a^		*Total (*n=*537)*^a^	
	N	%	N	%	N	%
	*Mean*	*S.d.*	*Mean*	*S.d.*	*Mean*	*S.d.*
	*Median*	*IQR*	*Median*	*IQR*	*Median*	*IQR*
*Sociodemographics*						
Age (years)						
Median (IQR)	60	48.9–67.1	59.1	48.1–66.1	59.4	48.7–66.8
Gender						
Male	95	35.20%	89	33.30%	184	34.30%
Female	175	64.80%	178	66.70%	353	65.70%
Ethnic origin (*n*=534)						
White	255	95.20%	252	94.70%	507	94.90%
Black/mixed	5	1.90%	5	1.90%	10	1.90%
Asian/mixed	6	2.20%	6	2.30%	12	2.20%
Other	2	0.80%	3	1.10%	5	0.90%
Highest level of education (*n*=505)						
No qualification/GCSE	88	34.70%	88	35.10%	176	34.80%
Vocational						
Vocational qualification/A-Level	69	27.20%	86	34.30%	155	30.70%
Degree or higher	91	35.80%	75	29.90%	166	32.90%
Other	6	2.40%	2	0.80%	8	1.60%
Deprivation (IMD) quintiles (*n*=526)						
1—Most deprived	18	6.70%	11	4.30%	29	5.50%
2	54	20.20%	45	17.40%	99	18.80%
3	77	28.80%	83	32.10%	160	30.40%
4	66	24.70%	49	18.90%	115	21.90%
5—Least deprived	52	19.50%	71	27.40%	123	23.40%
*Clinical*						
Weight (*n*=536) (kg)						
Mean (S.d.)	101.2	−17.5	100.4	−17	100.8	−17.2
Median (IQR)	98.6	88.4–110.7	97.6	88.4–108.3	98.4	88.4–109.7
BMI (*n*=536) (kg m^−^^2^)						
Median (IQR)	34.8	32.6-39.4	35	32.6-38.7	35	32.6-39.2
Waist (*n*=534) (cm)						
Median (IQR)	112	104–118	111.3	103–120	111.5	104–119
Blood pressure (mmHg						
Systolic (*n*=532), mean (s.d.)	136.6	−16.4	136.5	−17.5	136.5	−17
Diastolic (*n*=532), mean (s.d.)	81.4	−10.1	81	−10	81.2	−10.1
Cholesterol (mg dl^−1^)						
Total (*n*=473), mean (s.d.)	5.2	−1.1	5.2	−1.2	5.2	−1.2
LDL (*n*=282), mean (s.d.)	2.9	−1	2.9	−0.9	2.9	−1
Glucose (n=470) (mmol l^−1^)						
Mean (s.d.)	5.9	−2.4	5.8	−2.1	5.8	−2.2

Abbreviations: BMI, body mass index; GCSE, General Certificate of Secondary Education; IMD, index of multiple deprivation; IQR, interquartile range; LDL, low-density lipoprotein.

^a^Unless otherwise stated.

All patients randomised to 10TT received the 10TT leaflet. Each PCP’s ‘usual care’ strategies for weight management are described in Table 2. The majority made a community referral (*n*=10) or referred people for ‘in-house’ lifestyle advice (*n*=9). Some patients reported following weight loss programmes over and above what they had been allocated to in the trial during the 3-month intervention period (usual care *n*=89, 33%; 10TT *n*=97, 36%) and at 24 months (usual care *n*= 42, 16%; 10TT *n*=39, 15%).

**Table 2:** Usual care options by PCP

*PCP*	*Usual care*
PCP 1	Lifestyle advice+monitoring (monthly appointments)+community referral (gym prescription: 12 weeks)
PCP 2	Lifestyle advice+referral to dietitian+monitoring (weekly appointments)
PCP 3	Referral to dietitian (minimum 2 appointments)
PCP 4	Referral to dietitian (minimum 2 appointments)
PCP 5	Community referral (Camden Weight Management: 12 weekly sessions)
PCP 6	Lifestyle advice+referral to dietitian/psychologist or community referral (Camden Active Health: 12 weekly sessions)
PCP 7	Community referral (Weight Watchers: 12 weekly sessions)
PCP 8	Community referral (Weight Watchers: 12 weekly sessions)
PCP 9	Lifestyle advice+monitoring+community referral (diet and exercise lifestyle trainer: minimum 2 appointments)
PCP 10	Lifestyle advice+monitoring (monthly appointments)
PCP 11	Lifestyle advice+community referral (diet club or gym: 12 weeks)
PCP 12	Lifestyle advice+referral to dietitian+community referral (Somerset Activity and Fitness Group: 12 weekly sessions)
PCP 13	Lifestyle advice+monitoring (6 months programme: weekly for 4 weeks, then monthly), community referral (diet club or gym: 12 weeks)
PCP 14	Lifestyle advice+community referral (Slimming World+gym prescription, 12 weeks)

Abbreviation: PCP, primary care physician.

At 3 months, 389 (72.4%) patients were followed up. Those who did not complete the 3-month assessment tended to be from more deprived residential areas (median IMD centile = 61 vs 53 for those who attended), younger (mean age of 54.7 vs 58.3 years) and had a lower glucose level (mean glucose 5.3 compared with 6.0). Attrition rates also differed by PCP. There was little difference in attrition rate between arms at 3 months (26.3% in the usual care group vs 28.4% in the 10TT group, *P*=0.51), nor in the reason for attrition (Fisher’s exact test *P*=0.83). Men were slightly more likely to drop out in the 10TT arm than in the usual care arm (*P*-value for interaction=0.038).

At 24 months, 312 (58.1%) patients were followed up. There remained very little difference in attrition between arms (41.5% in the usual care group vs 42.3% in the 10TT group).

### Primary outcome

Primary outcome data were available for 383 participants. At the 3-month follow-up, patients who received 10TT had a mean weight loss of 1.68 kg (s.d.=3.21) compared with 0.84 kg (s.d.=2.83) in the ‘usual care’ group. Results from the mixed linear model showed that patients receiving 10TT lost significantly more weight over 3 months than patients receiving usual care (mean difference: 0.87 kg; 95% confidence interval (CI): −1.47 to −0.27, *P*= 0.004).

### Sensitivity to missing outcome data for the primary outcome

Adjustment for predictors of missing values, multiple imputation and baseline observation carried forward all gave similar results, with a difference in weight loss between arms ⩾0.58 kg, and *P*-values ⩽0.01 (Table 3).

**Table 3:** Sensitivity analyses for missing primary outcomes

*Method*	*N*	*Mean weight change (kg)*		*Difference*	*95% CI*	P*-value*
		*Usual Care*	*10TT*			
Primary analysis	383	−0.84	−1.68	−0.87	−1.47, −0.27	0.004
Adjusted for baseline predictors of missingness^a^	378	−0.83	−1.67	−0.94	−1.55, −0.33	0.003
Multiple imputation^b^	537	−0.78	−1.8	−1.03	−1.77, −0.30	0.006
Baseline observation carried forward	536	−0.61	−1.18	−0.58	−1.02, −0.14	0.01

Abbreviations: CI, confidence interval; 10TT, Ten Top Tips.

^a^Baseline weight, age, gender, deprivation and glucose level.

^b^Multiple imputation model including baseline sociodemographics and anthropometrics data, and other baseline variables possibly related to missingness and outcome. Imputations performed stratified by trial arm. Number of imputations=100.

We performed a range of sensitivity analyses assuming non-responders had a lower weight loss compared with those who completed the study follow-up (MNAR). Although the exact mean difference estimate varied, the direction of the effect was robust to a wide range of scenarios (see Supplementary Material).

### Secondary outcomes at 3 months

Changes in BMI and waist circumference at 3 months were in line with changes in weight (Table 4). More patients who received 10TT achieved at least 5% weight loss at 3 months (16%), compared with 8% in the usual care group (odds ratio (OR)=2.16; 95% CI=1.14, 4.12). There was little difference in clinical markers for potential comorbidities, with the exception of systolic blood pressure, which dropped by 3.59 mm Hg in the intervention group compared with 0.97 mm Hg in the usual care group (adjusted difference=2.98; 95% CI=−5.73, −0.23). There was a larger increase in the automaticity of the target behaviours within the intervention group compared with the control group (adjusted difference=8.45; 95% CI =2.59, 14.32).

**Table 4:** Secondary outcomes over 24 months

*Outcome*	*3 months*		*6 months*		*12 months*		*18 months*		*24 months*	
	N	%	N	%	N	%	N	%	N	%
	*Mean*	*S.d.*	*Mean*	*S.d.*	*Mean*	*S.d.*	*Mean*	*S.d.*	*Mean*	*S.d.*
*5% reduction in body weight*										
Control										
No	180	92	150	86	108	71	87	69	110	74
Yes	16	8	24	14	44	29	40	32	39	26
Intervention										
No	157	84	125	75	107	75	97	77	105	73
Yes	30	16	41	25	36	25	29	23	38	27
*Change in BMI*										
Control	−0.3	−1.03	−0.61	−1.4	−0.8	−1.78	−1.24	−2.84	−1.06	−2.68
Intervention	−0.6	−1.12	−0.83	−1.76	−0.8	−1.98	−0.78	−1.92	−0.72	−2.16
*Change in waist circum (cm)*										
Control	−1.88	−6.1	−1.61	−6.55	−2.3	−8.96	−2.31	−9.28	−2.33	−8.44
Intervention	−2.64	−6.03	−2.77	−7.22	−1.78	−7.23	−2.01	−7.97	−2.66	−7.58
*Change in systolic blood pressure*										
*Control*	−0.97	−16.15	—	*—*	*—*	*—*	*—*	*—*	*—*	*—*
*Intervention*	−3.59	−15.9	*—*	*—*	*—*	*—*	*—*	*—*	*—*	*—*
*Change in diastolic blood pressure*										
Control	−2.7	−9.84	—	—	—	—	—	—	—	—
Intervention	−2.61	−10.5	—	—	—	—	—	—	—	—
*Change in total blood cholesterol*										
Control	−0.21	−0.8	—	—	—	—	—	—	—	—
Intervention	−0.12	−0.74	—	—	—	—	—	—	—	—
*Change in LDL cholesterol*										
Control	−0.14	−0.62	—	—	—	—	—	—	—	—
Intervention	−0.07	−0.53	—	—	—	—	—	—	—	—
*Change in glucose level*										
Control	0.06	−2.85	—	—	—	—	—	—	—	—
Intervention	0	−1.79	—	—	—	—	—	—	—	—
*Change in automaticity*										
Control	19.5	21	—	—	—	—	—	—	—	—
Intervention	26.9	22.4	—	—	—	—	—	—	—	—

Abbreviations: BMI, body mass index; LDL, low-density lipoprotein.

### Secondary outcomes over 24 months

At 24 months, patients who received 10TT had a mean weight loss of 2.15 kg (s.d.=5.75) and those who received usual care had lost 2.96 kg (s.d.=7.16). Results from the mixed-effects model showed a significant group × time interaction (*χ*^2^=10.79, d.f.=4, *P*=0.029). Therefore, separate models were fitted to examine weight loss at each time point (6, 12, 18 and 24 months). The results suggested that while weight loss in the usual care group was slower in the first 6 months, it continued until 18 months, whereas the 10TT group experienced a larger weight loss in the first 6 months, but did not lose any additional weight after this (Table 5 and Supplementary Material). Changes in BMI and waist circumference over the 24-month period were generally in line with changes in weight. At 24 months, 27% (*n*=73) of the 10TT group had achieved at least 5% weight loss, and 26% (*n*=74) had done so in the usual care group.

**Table 5:** Weight loss from baseline at each time point, by arm

*Time*	N	*Usual care*		*10TT*		Adjusted mean difference*	*95% CI*
		*Change from baseline*	*S.d.*	*Change from baseline*	*S.d.*		
3	383	−0.84	−2.83	−1.68	−3.2	−0.87	(−1.47, −0.27)
6	336	−1.64	−3.83	−2.54	−5.04	−0.88	(−1.82, 0.06)
12	292	−2.32	−5.03	−2.36	−5.48	−0.06	(−1.25, 1.13)
18	252	−3.29	−7.63	−2.05	−5.04	1.18	(−0.41, 2.77)
24	290	−2.96	−7.16	−2.15	−5.75	0.75	(−0.73, 2.24)

Abbreviations: CI, confidence interval; 10TT, Ten Top Tips. *Adjusted for baseline weight and site.

### Sensitivity to missing outcome data over 24 months

Adjusting for predictors of missingness and multiple imputation had little effect on the weight change results at 24 months (estimated mean difference in weight change=1.11 (95% CI: −0.42, 2.63) and 0.85 (95% CI: −0.59, 2.29), respectively). The overall trend using imputed values mirrored what we found using unimputed data, although the mean difference at 18 months was reduced (estimated mean difference in weight change=0.54; 95% CI: −0.86, 1.93).

### Basic costs

The cost of 10TT was typically around £23 ($32) per participant, comprising the cost of materials (logbook, wallet sized food label guide, 10TT leaflet; ~£3 ($4) per participant) plus an initial consultation with a nurse (£20 ($28) for 30 min consultation).^37^

## Discussion

Eating and activity behaviours are often called ‘habits’, and habits are frequently mentioned by weight management programmes. However, it is rare to see the habit model used formally. This is the first intervention explicitly based on habit-formation theory to be delivered in the primary care context and importantly the first evaluation of a simple weight loss advice leaflet. Our primary outcome was weight loss at 3 months, and we found that that the 10TT leaflet led to significantly more weight loss than usual care, with twice as many patients achieving at least 5% weight loss as in the usual care arm. In line with the changes in weight, patients who received 10TT also had a reduction in waist circumference. Most clinical markers did not appear to change significantly, which is unsurprising given that weight loss was modest, and with the relatively short follow-up of 3 months. Nevertheless, there was a small but notable drop in systolic blood pressure that could be related to loss in weight. Furthermore, patients who received 10TT reported a greater increase in automaticity of the target behaviours, which suggests that 10TT was more effective at establishing new habits by the end of the intervention period.

We also explored maintenance of weight loss over 24 months. Patients who had received 10TT maintained the weight loss achieved at 3 months at 2 years, with over a quarter (27%) achieving at least 5% weight loss. This is promising given that most weight loss studies see weight regain post-treatment.^38, 39, 40, 41^ However, at 24 months there was no longer a difference in the amount of weight lost between the 10TT and usual care groups. This may reflect the inclusion of a usual care comparator rather than a ‘no-treatment’ control group, particularly as in the majority of cases, usual care included a referral to a commercial programme, and these are typically effective for weight loss.^20^ The early increase in weight loss in the 10TT arm may also simply reflect a novelty effect which rapidly dissipates. On the other hand, it may reflect the immediate delivery of the 10TT as opposed to a delayed receipt of usual care (due to this being a referral that would require additional appointments, or joining a commercial weight loss group). Our study is limited in the fact that we do not have clear data on the uptake of the various usual care strategies post-referral. Participants may also have seen participation as a first step, which motivated them to go on to other weight management programmes, but the number of patients who reported following weight loss programmes over and above what they had been allocated to in the trial was similar across the two arms, both during the 3-month intervention period and at 24 months.

The amount of weight lost in our 10TT arm was smaller than that observed in the previous exploratory study.^26^ This is not surprising given the likely increased heterogeneity of our population who were selected from PCP databases, compared with a volunteer population. The delivery of the leaflet and explanation of the habit model by primary care nurses rather than a research psychologist could also have a role. A recent systematic review highlighted that surprisingly few trials have tested weight loss interventions in everyday delivery settings by the actual practitioners who would deliver such interventions in routine practice.^42^ A strength of this study is that it was carried out within the primary care setting by health-care professionals across England, enhancing generalisability, and increasing the potential for the findings to inform clinical practice.

This is also the first RCT to our knowledge to demonstrate the effectiveness of a leaflet for weight loss. Leaflets are typically used as minimal intervention control arms in studies testing the effectiveness of more intensive weight management programmes. A recent examination of the effectiveness of such minimal intervention control groups across 29 studies found a mean weight change of −0.8 kg at 12 months.^43^ The 10TT leaflet achieved three times this (−2.4 kg at 12 months), which is notable for a low-intensity intervention. An additional advantage of 10TT is that the cost of the intervention is low, typically around £23 ($32) per participant. This appears cheaper than other primary care led or commercial weight management programmes.^20^ However, a full cost effectiveness analysis is under preparation, accounting for the impact on use of health services and medications.

The weight loss observed in the 10TT arm may reflect a change in those behaviours targeted, and the maintenance of this weight loss could reflect the fact that these behaviours have become habits. However, 10TT also uses self-monitoring and encourages regular self-weighing, both of which have been found to be effective in supporting weight loss.^44, 45^ It also included guidance on food labels. Patients reported that target behaviours had become more automatic at the end of the intervention period, but a more thorough process evaluation is required to improve our understanding of the mechanisms through which 10TT promotes weight loss.

Because of the nature of our intervention, it was not possible to blind participants; however all follow-up assessments for the primary outcome were with a health professional blind to treatment allocation, and were objective rather than self-reported. Although attempts were made to minimise contamination, health professionals delivering 10TT may have used the philosophy of the 10TT leaflet in the provision of care to patients randomised to usual care, although these patients would not have received the actual materials. Loss to follow-up was similar to other weight management trials in primary care at 3 months^19, 20, 46, 47, 48, 49^ and lower than in other trials at 2 years.^41^ Good follow-up rates reduce the likelihood of bias from attrition, and the results remained essentially unchanged using various models to account for this missing data.

A comparison of our trial participants with adults with obesity in the 2011 HSE data^50^ demonstrates that they had a similar BMI (35 vs 34.5), but were slightly older (59 vs 53), and more were women (66% vs 57%), although men were still better represented than in many other weight management studies.^47, 48, 49, 51^ A limitation of our study is that people from the most deprived quintile of residence areas were under-represented (6% from the most deprived in our trial vs 20% in the English population), as were those with no educational qualifications (35% in our trial vs 50% in the population).

Because of the opt-in nature of the study, our participants are likely to have been more motivated than the wider population suffering from obesity. This has implications for the generalisability of our results, and it could be argued that these motivated individuals may have lost weight without intervention. On the other hand, models of behaviour change highlight that motivation alone does not always translate to action, and giving people the skills and support to act on their motivation is an important component of behaviour change.^52^ The participants in this study were interested in receiving support with their desire to lose weight, and 10TT could facilitate this within primary care. The brevity and low cost of 10TT could also encourage providers to offer this support so that more patients receive it. It should also be noted that the invitation itself may have contributed to participants’ motivation that might otherwise have been lacking. An evaluation of 10TT within a cluster randomised controlled trial would give a better indication of acceptability and effectiveness of 10TT across a whole practice population, and not just among those who are already motivated.

Our findings suggest that 10TT, a novel but simple habit-based intervention, is effective when delivered through primary care. The latest obesity guidance^4, 5^ suggests that a loss of just 3% of bodyweight may improve long-term health outcomes for the population suffering from obesity, if this weight loss is maintained. Over a quarter of patients who received 10TT achieved at least 5% weight loss at 2 years. Furthermore, 10TT was more effective than usual care for weight loss in the short term, and was as effective as usual care for weight loss over 2 years. Given the low intensity and limited cost of 10TT, and the fact that weight loss was maintained, it has the potential to fill the gap in weight management advice that can be delivered by the primary care team with minimal time and resource. For some patients, this support may be seen as a first step that could lead on to more intensive services, whereas for others, a brief intervention may be sufficient to promote sustained modest weight loss, and may be an alternative option for those patients for whom a commercial referral is either impractical or unappealing.

## Supplementary information

Supplementary Figure 1 (JPG 67 kb)

Supplementary Information (PDF 366 kb)

Supplementary Information (DOCX 20 kb)

Supplementary Figure 1 (PPT 989 kb)

Supplementary Information (DOCX 31 kb)
