# Correction to: A brief intervention for weight control based on habit-formation theory delivered through primary care: results from a randomised controlled trial

**DOI:** 10.1038/s41366-021-00862-x

**Published:** 2021-06-07

**Authors:** R. J. Beeken, B. Leurent, V. Vickerstaff, R. Wilson, H. Croker, S. Morris, R. Z. Omar, I. Nazareth, J. Wardle

**Affiliations:** 1 Department of Epidemiology and Public Health, University College London, London, UK; 2 Department of Primary Care and Population Health, University College London, London, UK; 3 Department of Statistical Science, University College London, London, UK

Correction to: *International Journal of Obesity*

10.1038/ijo.2016.206

The original version of this article unfortunately contained a mistake in Table 1. In the paper the authors report cholesterol as mg dl-1 (Table 1) however, the correct unit should be mmol/l. Glucose should be the same. The authors apologize for the error. The correct Table 1 can be found below.

**Table 1:** Baseline characteristics.

	Usual care (*n* = 270)^a^		10TT (*n* = 267)^a^		Total (*n* = 537)^a^	
	*N*	%	*N*	%	*N*	%
	Mean	S.d.	Mean	S.d.	Mean	S.d.
	Median	IQR	Median	IQR	Median	IQR
Socio-demographics						
Age (years)						
Median (IQR)	60	48.9–67.1	59.1	48.1–66.1	59.4	48.7–66.8
Gender						
Male	95	35.20%	89	33.30%	184	34.30%
Female	175	64.80%	178	66.70%	353	65.70%
Ethnic origin (*n* = 534)						
White	255	95.20%	252	94.70%	507	94.90%
Black/Mixed	5	1.90%	5	1.90%	10	1.90%
Asian/Mixed	6	2.20%	6	2.30%	12	2.20%
Other	2	0.80%	3	1.10%	5	0.90%
Highest level of education (*n* = 505)						
No qualification/GCSE	88	34.70%	88	35.10%	176	34.80%
Vocational qualification/A-Level	69	27.20%	86	34.30%	155	30.70%
Degree or higher	91	35.80%	75	29.90%	166	32.90%
Other	6	2.40%	2	0.80%	8	1.60%
Deprivation (IMD) quintiles (*n* = 526)						
1—Most deprived	18	6.70%	11	4.30%	29	5.50%
2	54	20.20%	45	17.40%	99	18.80%
3	77	28.80%	83	32.10%	160	30.40%
4	66	24.70%	49	18.90%	115	21.90%
5—Least deprived	52	19.50%	71	27.40%	123	23.40%
Clinical						
Weight (*n* = 536) (kg)						
Mean (S.d.)	101.2	−17.5	100.4	−17	100.8	−17.2
Median (IQR)	98.6	88.4–110.7	97.6	88.4–108.3	98.4	88.4–109.7
BMI (*n* = 536), kg/m^2^						
Median (IQR)	34.8	32.6–39.4	35	32.6–38.7	35	32.6–39.2
Waist (*n* = 534), cm						
Median (IQR)	112	104–118	111.3	103–120	111.5	104–119
Blood pressure (mmHg)						
Systolic (*n* = 532), mean (s.d.)	136.6	−16.4	136.5	−17.5	136.5	−17
Diastolic (*n* = 532), mean (s.d.)	81.4	−10.1	81	−10	81.2	−10.1
Cholesterol, mmol/l						
Total (*n* = 473), mean (s.d.)	5.2	−1.1	5.2	−1.2	5.2	−1.2
LDL (*n* = 282), mean (s.d.)	2.9	−1	2.9	−0.9	2.9	−1
Glucose (*n* = 470), mmol/l						
Mean (s.d.)	5.9	−2.4	5.8	−2.1	5.8	−2.2

*BMI* Body Mass Index, *GCSE* General Certificate of Secondary Education, *IMD* Index of Multiple Deprivation, *IQR* interquartile range, *LDL* Low-density lipoprotein.

^a^Unless otherwise stated.

