# Supplementary figures and images for: A brief intervention for weight control based on habit-formation theory delivered through primary care: results from a randomised controlled trial
Source: Int J Obes (Lond). 2016 Nov 21;41(2):246–54. doi: 10.1038/ijo.2016.206 (PMC5300101; doi:10.1038/ijo.2016.206)

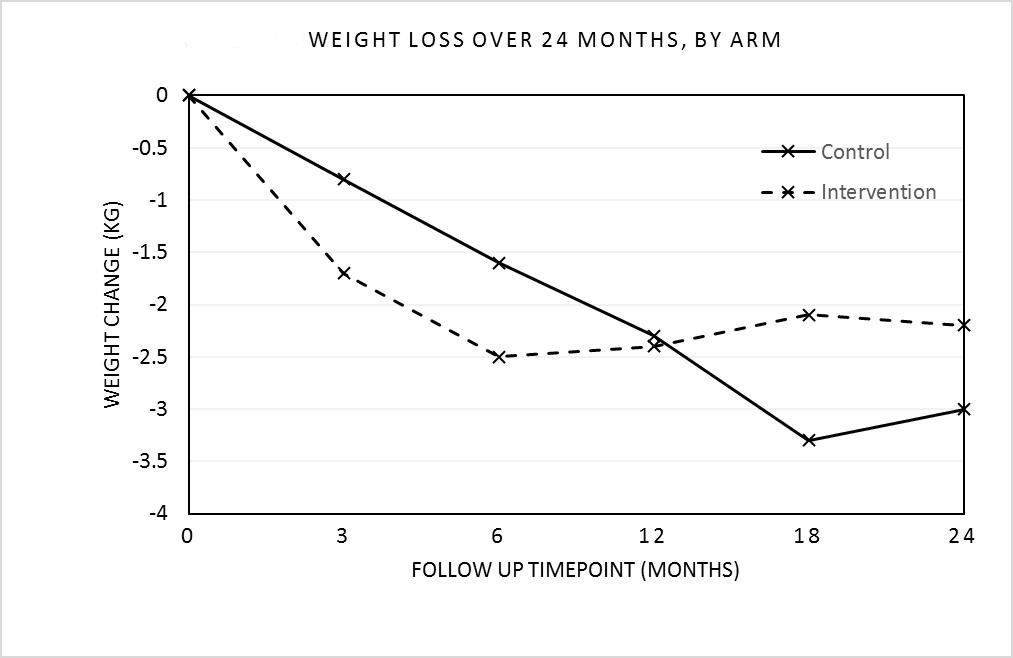

Supplement: Supplementary file 1 — Supplementary Figure 1 (JPG 67 kb) [file 41366_2017_BFijo2016206_MOESM7_ESM.jpg]
