# Supplementary material for: A brief intervention for weight control based on habit-formation theory delivered through primary care: results from a randomised controlled trial
Source: Int J Obes (Lond). 2016 Nov 21;41(2):246–54. doi: 10.1038/ijo.2016.206 (PMC5300101; doi:10.1038/ijo.2016.206)
Supplement: Supplementary file 2 — Supplementary Information (PDF 366 kb) [file 41366_2017_BFijo2016206_MOESM8_ESM.pdf]

reduce the risk

CANCER RESEARCH UK

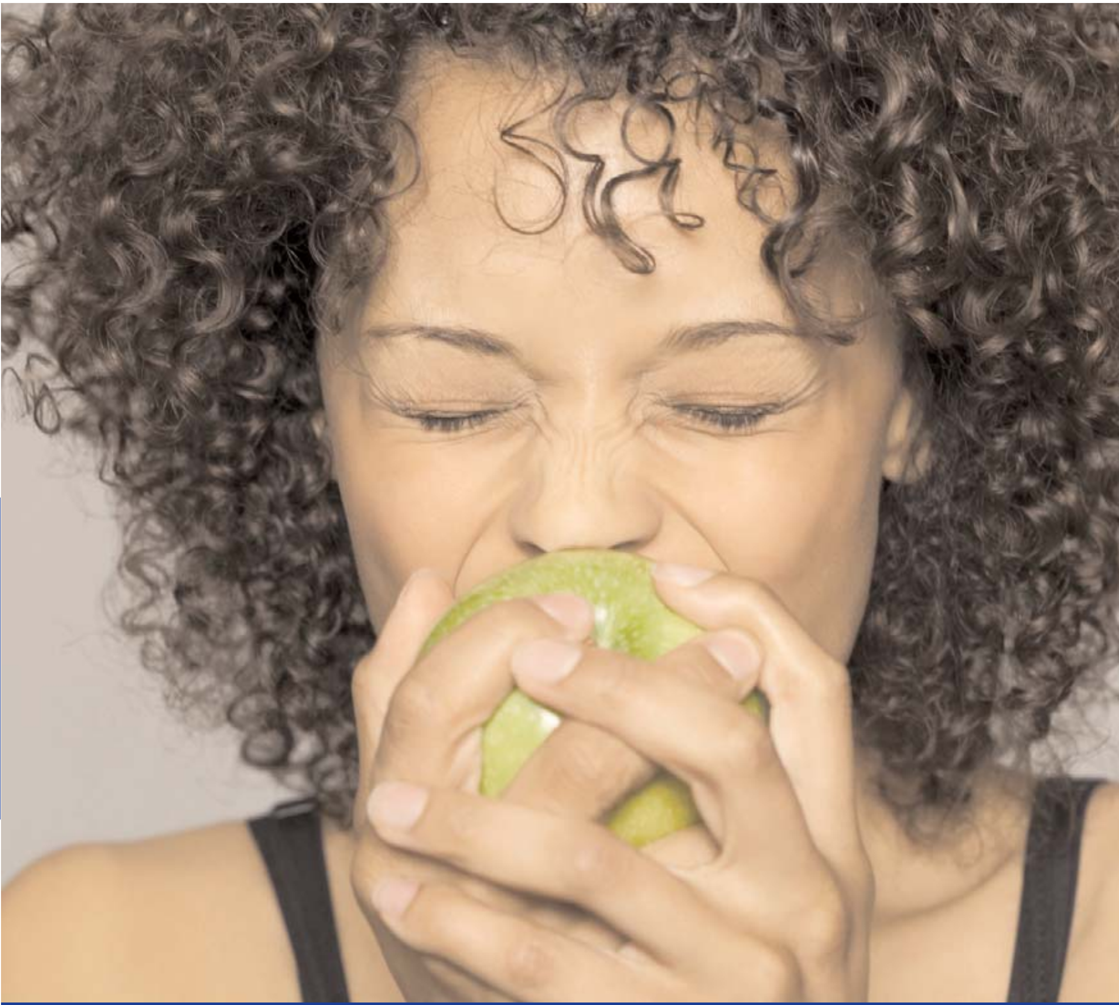

ten top tips

weight loss tips based on scientific evidence

in partnership with WEIGHTCONCERN

FIGHTING OBESITY WITH KNOWLEDGE

ten top **tips**

This leaflet contains a programme of weight loss tips, all based on scientific evidence. They will help you take in fewer calories and burn more energy through activity. Ten Top Tips are simple habits that everyone can fit into their daily routines and doing all ten over the longer-term will help you lose weight and keep it off.

How can the Ten Top Tips help you control your weight?

The Ten Top Tips programme helps you incorporate lifestyle changes into your daily routine so that they become automatic and easy to maintain. To develop new healthy habits you need to:

- **Plan ahead:** In the first week or two, spend a little time working out in advance how you are going to do the tips. For example work out how to fit five servings of fruit and vegetables and the extra walking into your day.
- **Tracking your progress:** Use tick sheets each day to record if you do each of the tips. Keep this up until the tips have become automatic. Record-keeping increases success in developing healthy habits.

A tear off tick sheet can be found at the back of this leaflet.

Does being overweight matter?

Being overweight affects your health. It can increase the risk of several types of cancer. These include cancers of the bowel, kidney, oesophagus (foodpipe) and womb, as well as breast cancer in women who have been through the menopause. It can also increase the risk of diabetes, high blood pressure, coronary heart disease, osteoarthritis and stroke.

We all know how difficult it is to lose weight and keep it off. The good news is that if you are overweight losing just 5-10% of your body weight and keeping it off will have a positive effect on your health. For most people this will be around 3-10kg or $^1/_2$ - $1^1/_2$ stone.

(NB If you are seriously overweight and have other health problems, it may be advisable to seek advice from your Doctor before beginning a weight management programme).

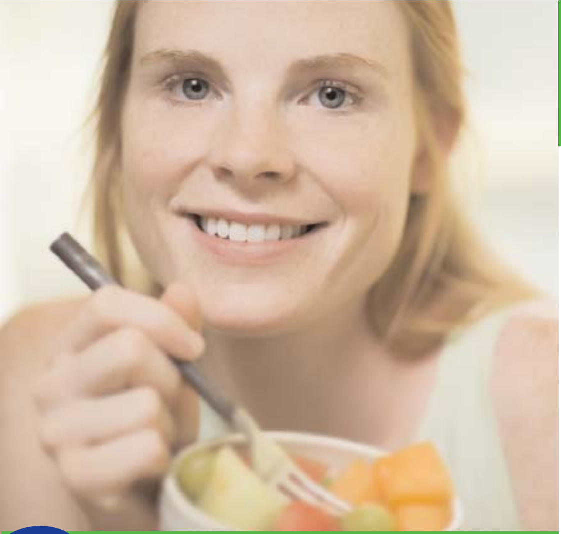

Keep to your meal routine

Try to eat at roughly the same times each day, whether this is two or five times a day.

Handy Hints:

- Pick a pattern that fits in with your own daily routine and stick to it.
- If you are someone who needs snacks, try to snack around the same time each day.
- Try planning when you intend to eat and check at the end of the day if you have achieved this.

2 Go reduced fat

Choose reduced fat versions of foods such as dairy products, spreads and salad dressings where you can. Use them sparingly as some can still be high in fat.

Handy Hint:

- Change to semi-skimmed milk and save 60 calories/day, amounting to 420 calories. over a week (based on consuming 300mls milk/day).

Keeping a record has been shown to increase people's success in developing **healthy habits**

3 Walk off the weight

Walk 10,000 steps (equivalent to 60-90 minutes moderate activity) each day. You can use a pedometer to help count the steps. You can break up your walking throughout the day.

Handy Hints:

- 5000 steps a day extra (40mins walking at a brisk pace), will burn 1240 calories over a week.
- Take the stairs rather than the lift.

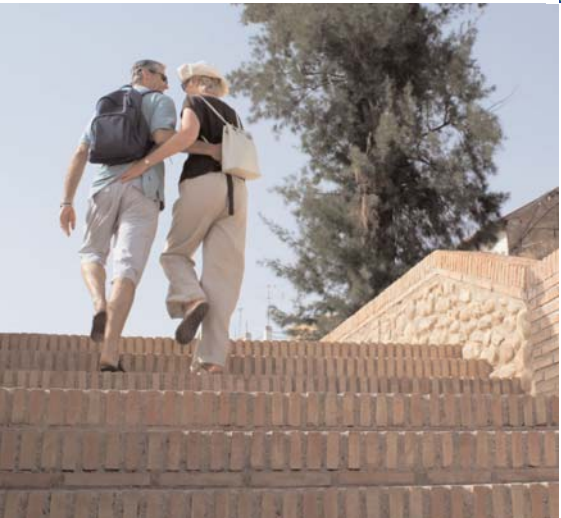

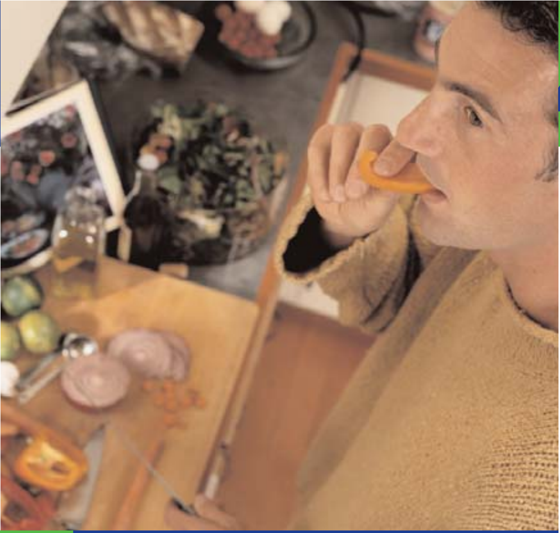

4 Pack a healthy snack

If you snack, choose a healthy option such as fresh fruit or low calorie yogurts instead of chocolate or crisps.

Handy Hints:

- Have a banana instead of a snack-size chocolate bar and save 225 calories.
- Take a piece of fruit to work in your bag so that you have it with you for a snack.
- Choose yogurts with less than 100 calories per pot. The calcium will also keep your bones healthy.

5 Look at the labels

Be careful about food claims. Check the fat and sugar content on food labels when shopping and preparing food.

Handy Hints:

- A low fat digestive biscuit has the same number of calories as the standard digestive biscuit at 70 calories. Check the fat and sugar content.
- Order our shopping guide from the Reduce the Risk website: www.reducetherisk.org.uk

Creating a **daily routine, keeping track of your progress** and **planning ahead** are key to developing healthier habits to last a lifetime.

6 Caution with your portions

Don't heap food on your plate (except vegetables). Think twice before having second helpings.

Handy Hint:

- Fill your plate up with lots of vegetables. They are low in calories and will help satisfy your hunger.

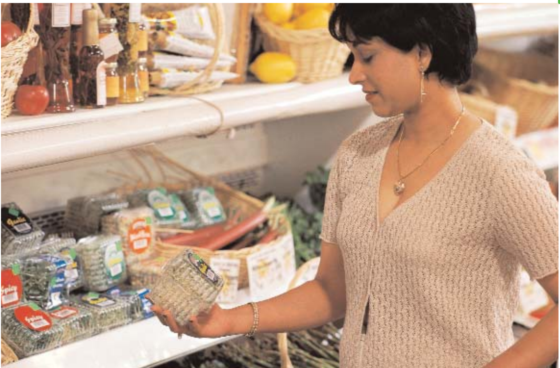

ten top tips tick sheet: Keeping track of your progress

Fill in this tick sheet every day to record whether or not you managed each tip. Keeping a record has been shown to increase people's success in developing healthy habits. Keeping track of your weight is also very useful. Daily weighing has been shown to increase successful weight control. In the notes column you can write details of how you are achieving the tips, and anything that particularly helps you use them. This information will help you plan for the next week.

ten top tips	m	t	w	t	f	s	s	done on 5 days or more?	notes
1. Keep to your meal routine									
2. Go reduced fat									
3. Walk off the weight (No. of steps)									
4. Pack a healthy snack									
5. Learn the labels									
6. Caution with your portions									
7. Up on your feet									
8. Think about your drinks									
9. Focus on your food									
10. Don't forget your 5 a day									
Your weight									

What do you plan to do next week? (e.g. I will write a shopping list to remind myself to buy fruit)

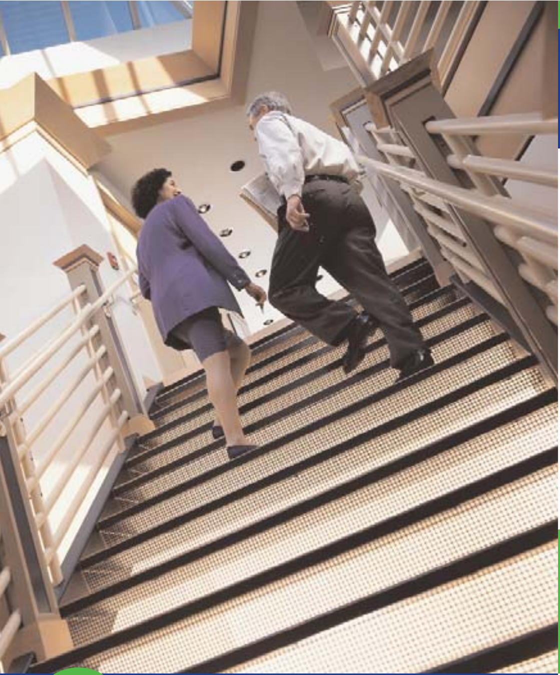

7 Up on your feet

Break up your sitting time. Stand up for ten minutes out of every hour.

Handy Hints:

- Standing up on the bus or train burns an extra 70 calories an hour.
- When watching TV try to stand up during the ad breaks and do a few chores (e.g wash the dishes or put the rubbish out).

8 Think about your drinks

Choose water or sugar-free squashes. Unsweetened fruit juice is high in natural sugar so limit it to 1 glass per day (200ml / $^{1}/_{3}$ pint). Alcohol is high in calories so try to limit the amount you drink.

Handy Hint:

- A pint of standard beer has 2 units of alcohol and 182 calories.

9 Focus on your food

Slow down. Don't eat on the go or while watching TV. Eat at a table if possible.

Handy Hints:

- Eating meals at the table will help you to focus on the amount of food you eat.
- Don't eat while walking, wait until you get there and take time to concentrate on what you are eating.

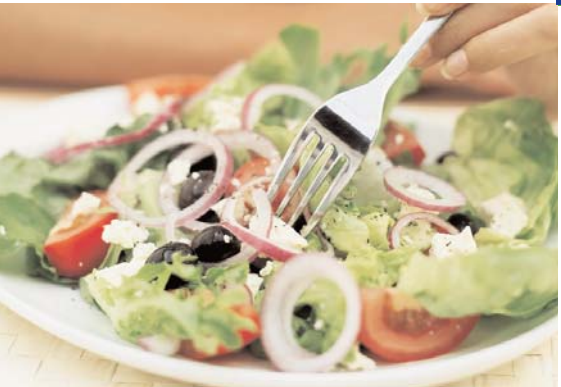

10 Don't forget your 5 a day

Eat at least 5 portions of fruit and vegetables a day (400g in total).

Handy Hints:

- A medium sized apple or banana or 3 serving spoonfuls of peas is 1 portion.
- Try having fruit or vegetables with every meal. This makes it easier to reach the five a day.

frequently asked **questions**

Should I choose 'diet' foods?

Low calorie foods can be useful for reducing your energy intake but be careful of misleading claims and check the labels. For example low fat biscuits and other sweet foods may be lower in fat but not in calories.

I don't eat any fruit and vegetables. Do I have to start eating five all at once?

It may work better for you to start eating a smaller number and build up over time. Make sure your plan is clear so you can judge if you achieve it or not each day.

Do I have to make all these changes at once?

The sooner you can build the tips into your life the quicker you'll start to lose weight. But it can be difficult to make all these changes at once. If you prefer you could try a smaller number until you feel confident enough to move on to others. The end goal is to fit all of the tips into your lifestyle.

The sooner you can **incorporate all the tips** into your life the quicker you'll start to lose weight.

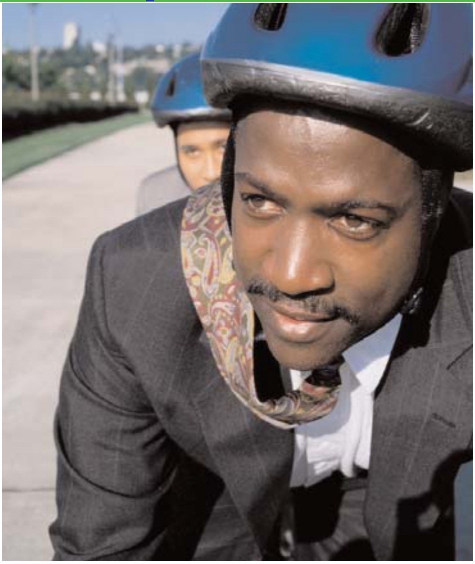

Should I avoid occasional opportunities to exercise if not part of my normal routine?

It is fine to do more but keep doing your routines as well. This applies to all the tips.

What do I do on days when I am doing something different to my normal routine? for example: when I am away on business or at weekends.

You can develop different routines for the doing the tips during the week and at weekends. If you are away on a trip, do your best to stick to most of the tips and then get back to your normal routine as soon as you return.

What if I lose interest in the tips?

Changing habits of a lifetime is hard work but once the tips become part of your normal routine you will hardly notice doing them. Just like brushing your teeth.

Guide to shopping

The following information provides you with some simple advice for understanding food labels.

	per 100g	
	a lot	a little
Sugars	10g	2g
Fat	20g	3g
Saturated fat	5g	1g
Fibre	3g	0.5g
Salt	1.25g	0.25g
Sodium	0.5g	0.1g

Reproduced with permission from the Foods Standards Agency

Look for the following information on food labels to make healthy choices:

Snacks
Less than 3g fat
and less than 8g sugar per serving

Breakfast cereals
Less than 5g fat
and less than 10g sugar per 100g

Ready meals
Less than 10g fat
and less than 350kcal per portion

Pre-packed sandwiches
Less than 6g fat
and less than 280kcal per sandwich pack

further information

Visit www.reducetherisk.org.uk for further information about the Ten Top Tips and more about the healthy choices that could reduce your risk of cancer.

Visit Weight Concern's website www.weightconcern.org.uk for further information about obesity and weight loss.

For more about cancer visit Cancer Research UK's patient information website www.cancerhelp.org.uk.

About **Cancer Research UK**
Cancer Research UK is the leading charity dedicated to research on the causes, treatment and prevention of cancer. If you would like to support our work please call 020 7121 6699 or visit our website.

Cancer Research UK
PO Box 123
London
WC2A 3PX
020 7242 0200
www.cancerresearchuk.org

About **Weight Concern**
Weight Concern is a UK charity committed to researching and developing more effective treatments for obesity. We also train health professionals in techniques to help support people who want to control their weight.

Weight Concern
Brook House
2-16 Torrington Place
London
WC1E 7HN
enquiries@weightconcern.org.uk
www.weightconcern.org.uk

Reg charity no. 1089464 2006 Ref: rtr400
