# Supplementary material for: A brief intervention for weight control based on habit-formation theory delivered through primary care: results from a randomised controlled trial
Source: Int J Obes (Lond). 2016 Nov 21;41(2):246–54. doi: 10.1038/ijo.2016.206 (PMC5300101; doi:10.1038/ijo.2016.206)
Supplement: Supplementary file 3 — Supplementary Information (DOCX 20 kb) [file 41366_2017_BFijo2016206_MOESM9_ESM.docx]

**Explanation of the 10 Top Tips Intervention**

If the patient has been allocated to receive the 10 Top Tips intervention, you will now talk them through the intervention.

Quite a lot of detail is provided in this section to ensure a standardized delivery of the intervention to patients.

Additional materials needed:

- Information cards
- Ten top tips leaflet
- Ten top tips logbook
- Example of additional information that will be available on the Weight Concern website (or ideally access to the internet) (Appendix 8).

You will have roughly 30 minutes to go through the intervention with the patients. Patients should already have a general understanding of ten top tips (see section on explaining the study, p.23)

Aims of this session

1. Ensure that patients have a thorough understanding of what the intervention is about and what they need to do.
2. Motivate patients to keep going with the intervention for the full three months.
   1. Factors that may help with this:
      1. The simplicity of the intervention
      2. The benefits of following the tips
      3. Following the tips over a longer period will make it more likely that healthy habits develop
3. Help patients to consider how the tips can be fitted into their lifestyle and work through any initial problems.

Overview

|  | Time (minutes) |
| --- | --- |
| Introduction and previous experience of weight management | 5 |
| Information cards 1-4 | 20 |
| Explain ten top tips leaflet |  |
| Cards 5-6 |  |
| Explain logbook |  |
| Cards 7-12 |  |
| Questions | 5 |

1. Introduction
   1. Explain that you will now have around 30 minutes to outline the ten top tips intervention in more detail. The purpose of this is to help them understand what they have to do, and give them the chance to ask any questions if they have uncertainties.
2. Previous experience of weight management
   1. Explain that, as mentioned earlier, ten top tips is about making healthy choices into habits to help them manage their weight. Ask patients whether they have tried to lose weight before. If so, what have they tried before?
      1. This will help you to estimate how responsive the patient will be to the leaflet and will help to develop rapport.
   2. Most patients will have tried to lose weight before and you might find it useful to follow it up with one of these statements:
      1. “You might find that you are already familiar with some of the healthy choices here and that means that you can get off to a good start”
      2. “You might find that this is a bit different to things you’ve tried before but it’s straightforward and we’ve got plenty of time now to go through it”.
      3. If patients haven’t tried to lose weight before you could respond with “Hopefully you’ll find that this is quite straightforward and we’ve got plenty of time to go through it now”.
3. Using the laminated cards (Allow up to 20 minutes)
   1. Say that you’re now going to go through some cards that explain the intervention in more detail.
   2. Read through cards 2 and 3 on weight gain and losing weight.
   3. After reading through card 4 about ten top tips, refer to the ten top tips leaflet.
   4. Show how the ten tips are laid out across the leaflet, briefly pointing out that there is some introductory information at the beginning, some frequently asked questions and extra information on the back and a monitoring form (which you will talk about in more detail later).
   5. Explain that the tips were
      1. Deliberately chosen to be simple so that they were easy for patients to follow and build into their life.
      2. Based on scientific evidence that shows they can help people lose weight.
      3. Some of them will help patients to take in fewer calories (e.g. ‘go reduced fat’ and ‘caution with your portions’), some of them will help them to be more active (‘walk off the weight’) and some will help them to be more aware of what they’re doing (e.g. focus on your food).
   6. Read through one tip in detail, including its handy hints.
   7. Return to the information cards and explain that a key part of this intervention is about practising the ten top tips until they become healthy habits.
   8. Talk through cards 5 and 6 (healthy habits and turning the tips into habits). When explaining how habits can affect our health on card 5, give an example of a positive habit as well e.g. always taking the stairs instead of the lift at work which increases fitness levels.
   9. Refer to the logbook. Explain that the log book will help them to turn the tips into habits. Write the patient code on the inside cover. Ask them to contact you if they lose this and you will send them a new one.
   10. Show the patient that there is a blank tick sheet and blank notes and planning sheet for each week. (They work as a double page spread). Refer to the filled in example pages at the start of the logbook and emphasise these points:
       1. **Fill in the tick boxes every day for each tip** (go vertically!). Explain that keeping track of how you are doing with the tips has been shown to increase the chances of forming healthy habits.
       2. **Use the space to record their weight regularly** (at least once a week, and no more than once a day). Tell the patient that research shows that people who monitor their weight regularly are more successful at losing weight and maintaining weight loss.
       3. **See if they’ve done the tip on 5 or more days**. This will help them to see which tips they are doing well with and which they need to work harder on.
       4. **Use the notes and planning sheet each week** to make notes of how they are doing and plan if they will do anything differently next week. Emphasise again that planning how you will do something increases the chances of you actually doing it.
       5. **Use a new double page spread in the logbook each week and keep going until their next appointment in three months**. Explain that it might seem like a long time but the longer they follow the tips, the more likely they will become habits that could last a lifetime. They can look back in the logbook over this time to see their progress. NB. Patients will be sent a pre-paid envelope to post the logbooks back after three months. This is for research purposes only and individuals will not identified by the logbooks.
   11. Return to the cards saying something along the lines of “So I thought we could have a go at thinking how you could fit these tips into your daily life?”
   12. Talk through the examples on cards 7 and 8, stating that for each tip, the patient will need to plan when and how it can be built into their daily routine.
   13. Ask the patient if they think they will be able to fit the tips into their routine and explain that you can have a go at planning one of the tips with them now if they like (card 9). It’s ok if they don’t want to do this.
   14. Say that you want to check that you’ve explained it clearly, so ask the patient if they can run you through what they have to do.
   15. Follow this up by reading through card 10 (recap) if you need to.
   16. Read through card 11. Before moving onto card 12, ask the patient what they hope to gain from following the ten top tips. Follow this up with card 12 (the benefits of following the tips). If patients ask further questions about weight loss it might be helpful to state that the maximum amount of weight loss per week (0.5kg or 1 pound) was estimated from someone doing none of the activities to all of activities. Depending on what patients are already doing, and how regularly they follow the tips, the weight loss might not be as much as this. You should emphasise that regardless of weight loss, increasing physical activity levels and following a healthier diet will bring benefits to their health.
   17. Ask if the patient has any final questions.

**Note: Suggest that they may like to buy a pedometer (most people enjoy using them) and advise them where they can do so, but let them know that they may record the amount of minutes spent walking rather than steps if they don’t have a pedometer.**
