# Supplementary material for: A brief intervention for weight control based on habit-formation theory delivered through primary care: results from a randomised controlled trial
Source: Int J Obes (Lond). 2016 Nov 21;41(2):246–54. doi: 10.1038/ijo.2016.206 (PMC5300101; doi:10.1038/ijo.2016.206)
Supplement: Supplementary file 4 — Supplementary Figure 1 (PPT 989 kb) [file 41366_2017_BFijo2016206_MOESM10_ESM.ppt]

## Slide 1
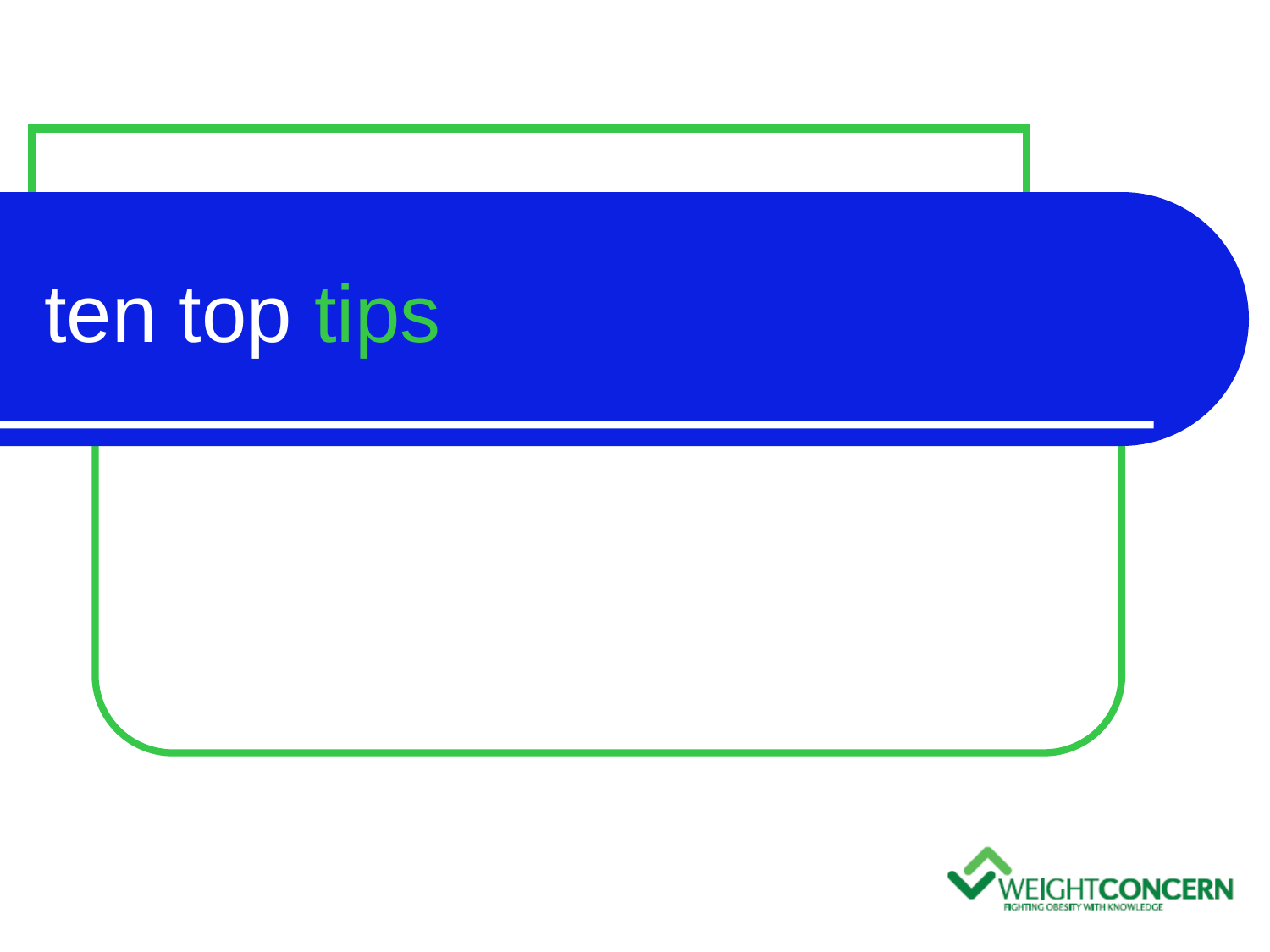

# ten top tips

## Slide 2
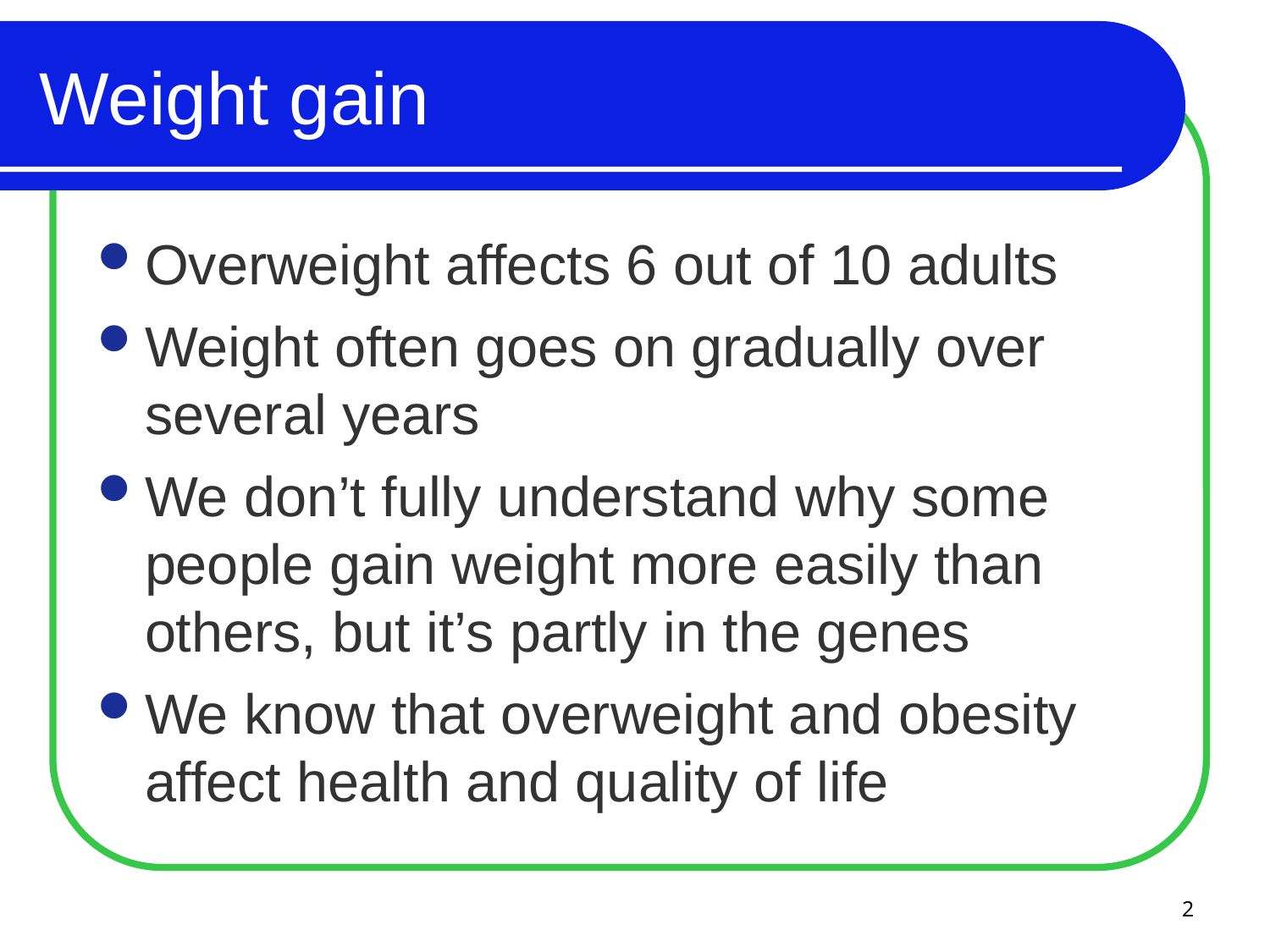

# Weight gain
Overweight affects 6 out of 10 adults
Weight often goes on gradually over several years
We don’t fully understand why some people gain weight more easily than others, but it’s partly in the genes
We know that overweight and obesity affect health and quality of life
<number>

## Slide 3
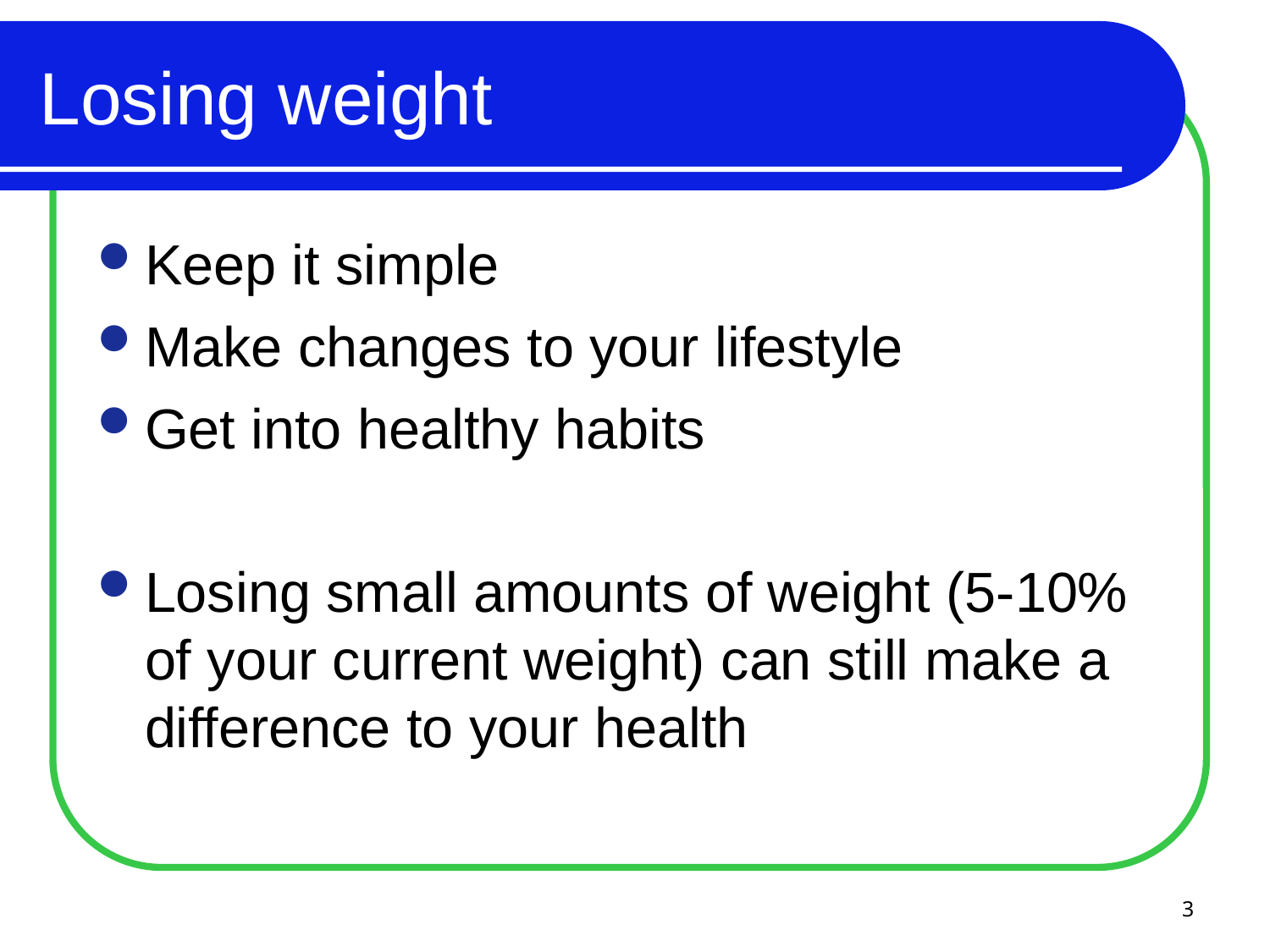

# Losing weight
Keep it simple
Make changes to your lifestyle
Get into healthy habits
Losing small amounts of weight (5-10% of your current weight) can still make a difference to your health
<number>

## Slide 4
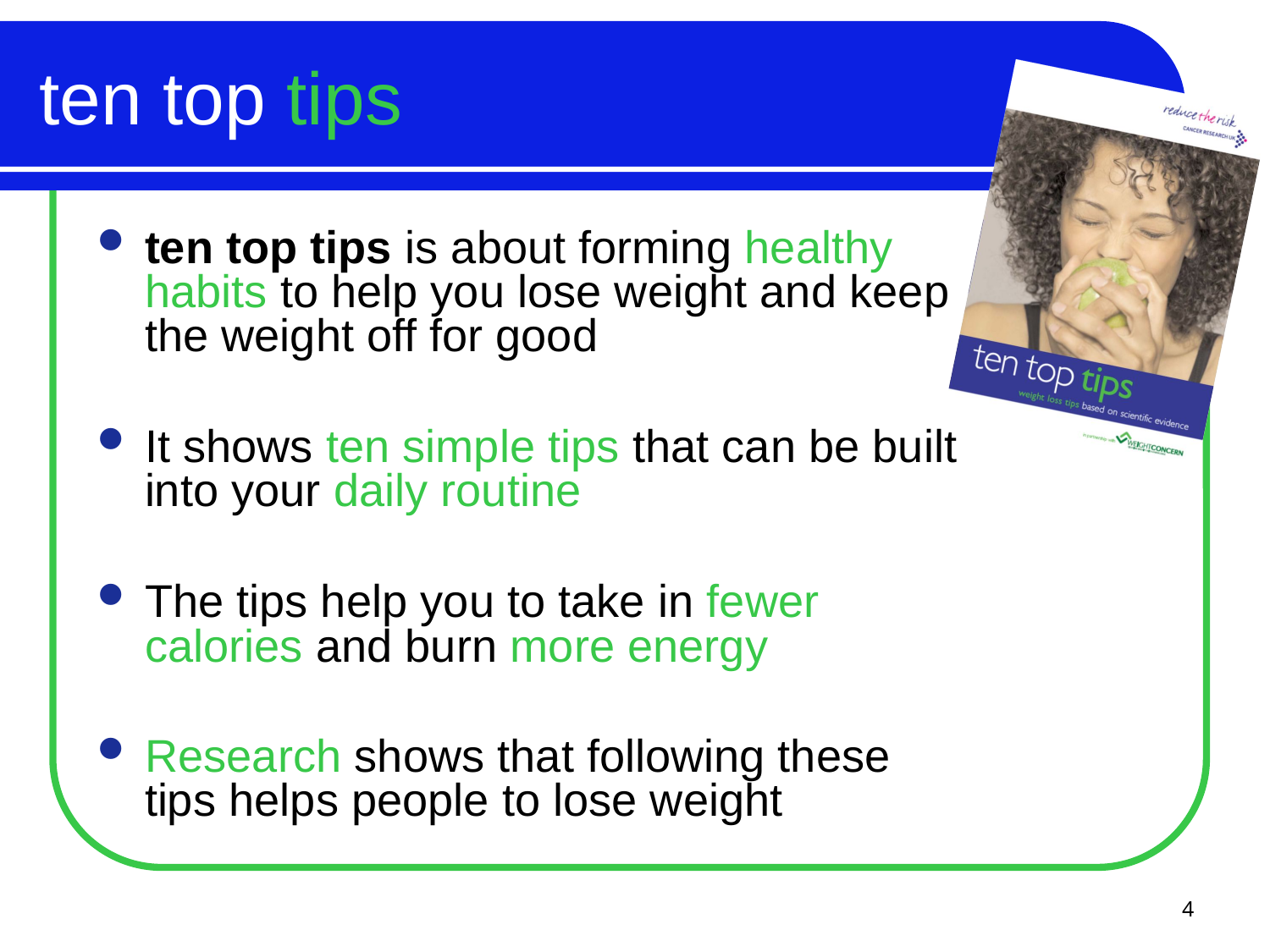

# ten top tips
ten top tips is about forming healthy habits to help you lose weight and keep the weight off for good
It shows ten simple tips that can be built into your daily routine
The tips help you to take in fewer calories and burn more energy
Research shows that following these tips helps people to lose weight
<number>

## Slide 5
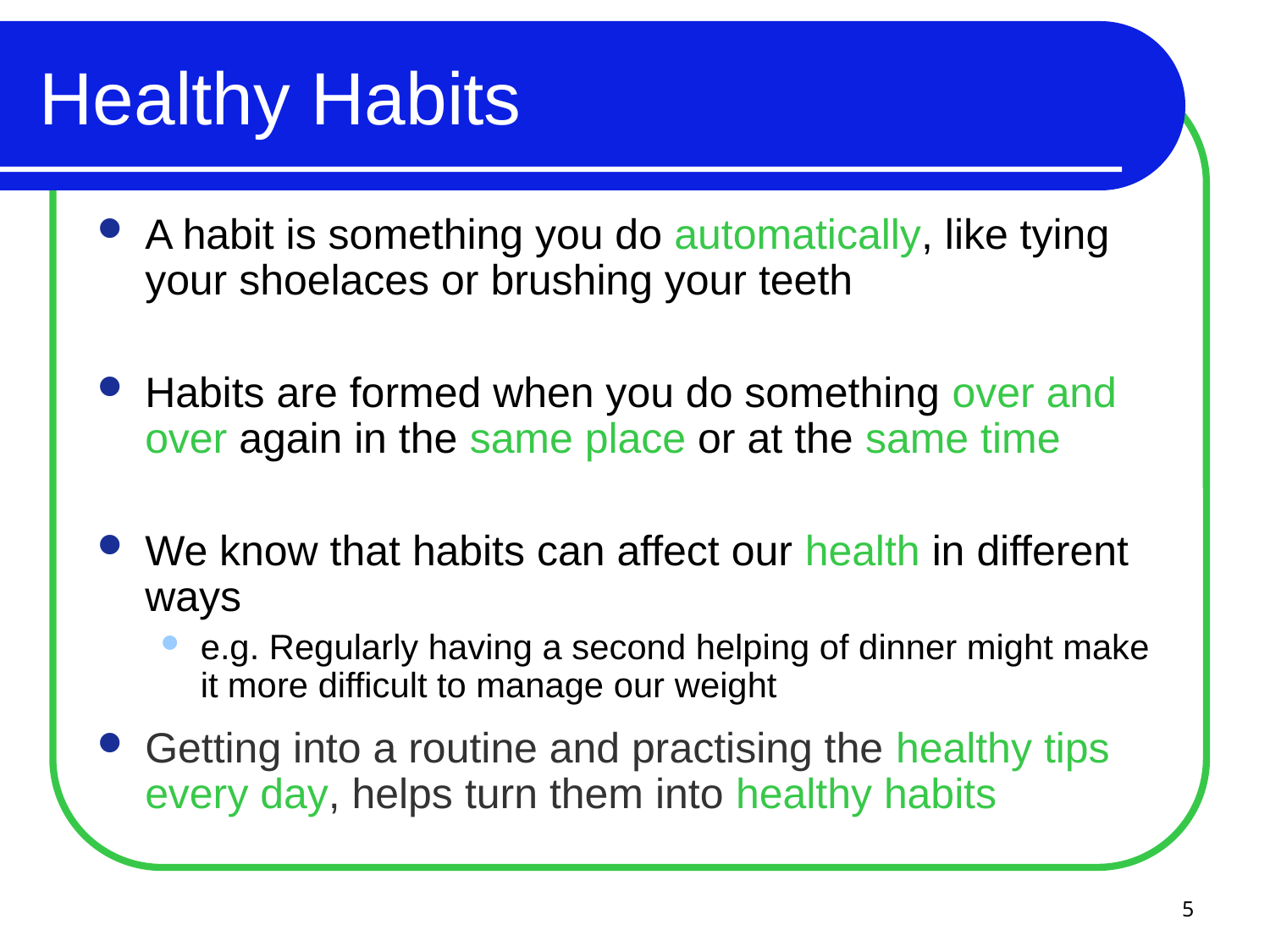

# Healthy Habits
A habit is something you do automatically, like tying your shoelaces or brushing your teeth
Habits are formed when you do something over and over again in the same place or at the same time
We know that habits can affect our health in different ways
e.g. Regularly having a second helping of dinner might make it more difficult to manage our weight
Getting into a routine and practising the healthy tips every day, helps turn them into healthy habits
<number>

## Slide 6
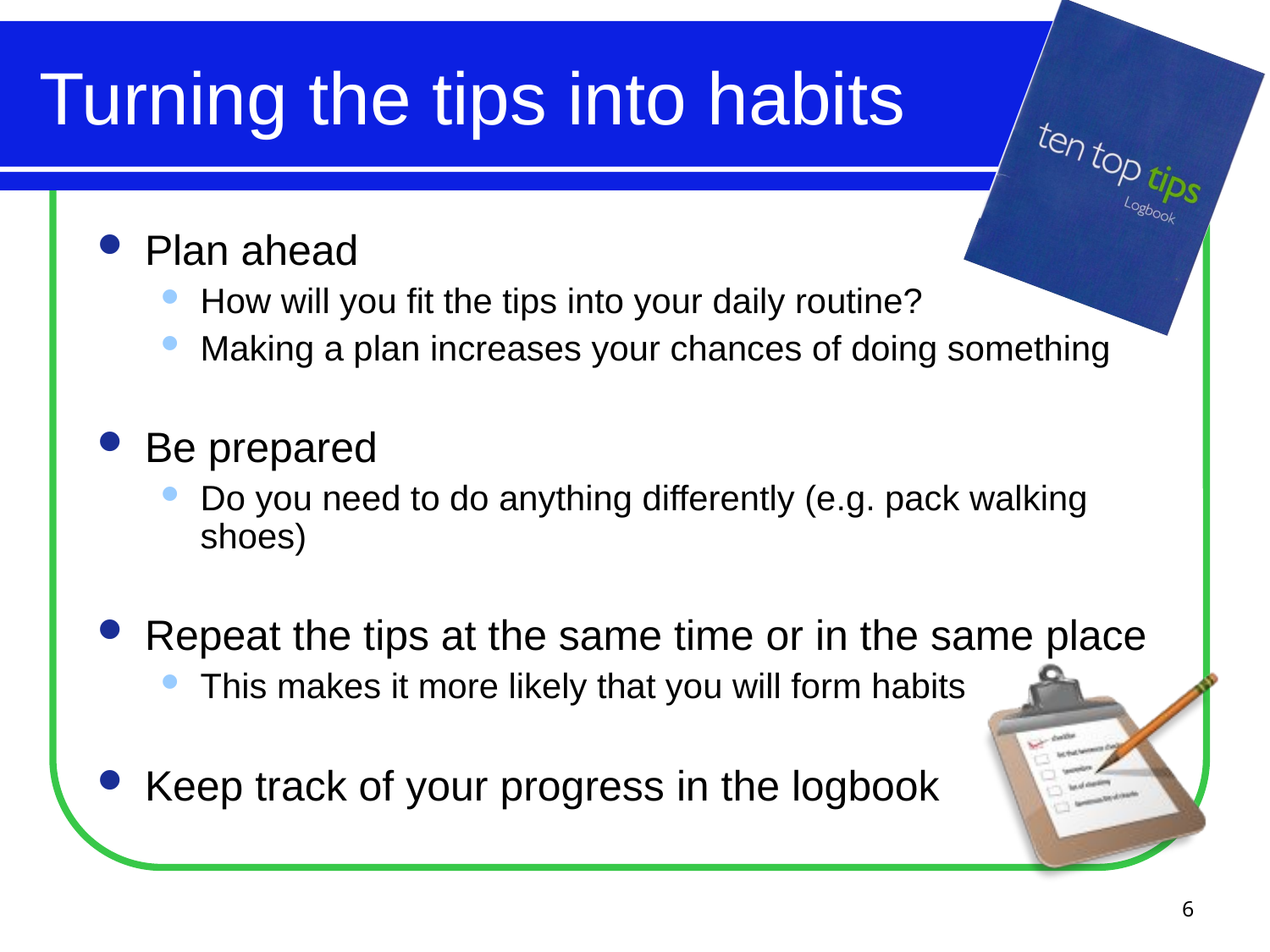

# Turning the tips into habits
Plan ahead
How will you fit the tips into your daily routine?
Making a plan increases your chances of doing something
Be prepared
Do you need to do anything differently (e.g. pack walking shoes)
Repeat the tips at the same time or in the same place
This makes it more likely that you will form habits
Keep track of your progress in the logbook
<number>

## Slide 7
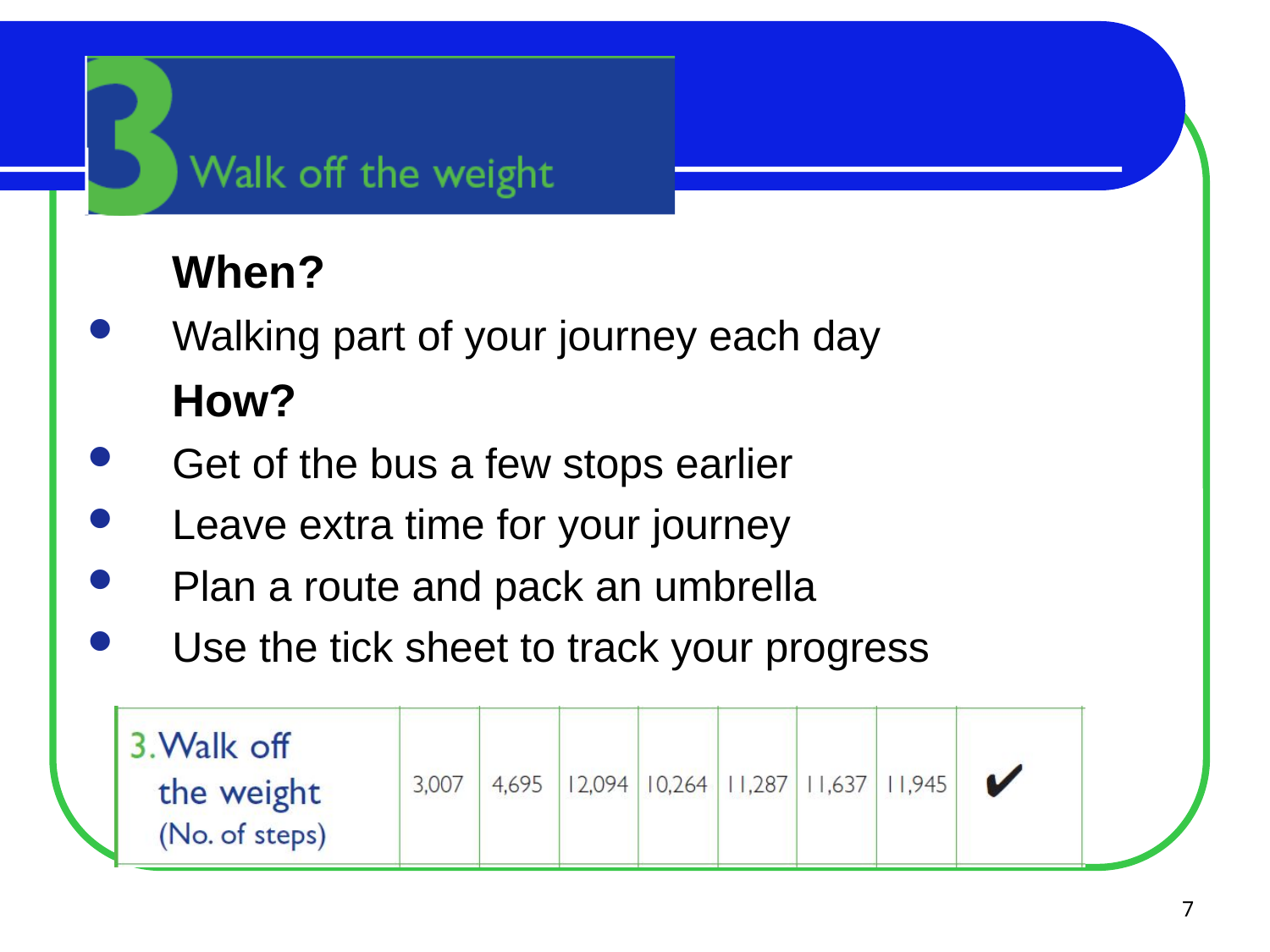

# When?
Walking part of your journey each day
How?
Get of the bus a few stops earlier
Leave extra time for your journey
Plan a route and pack an umbrella
Use the tick sheet to track your progress
<number>

## Slide 8
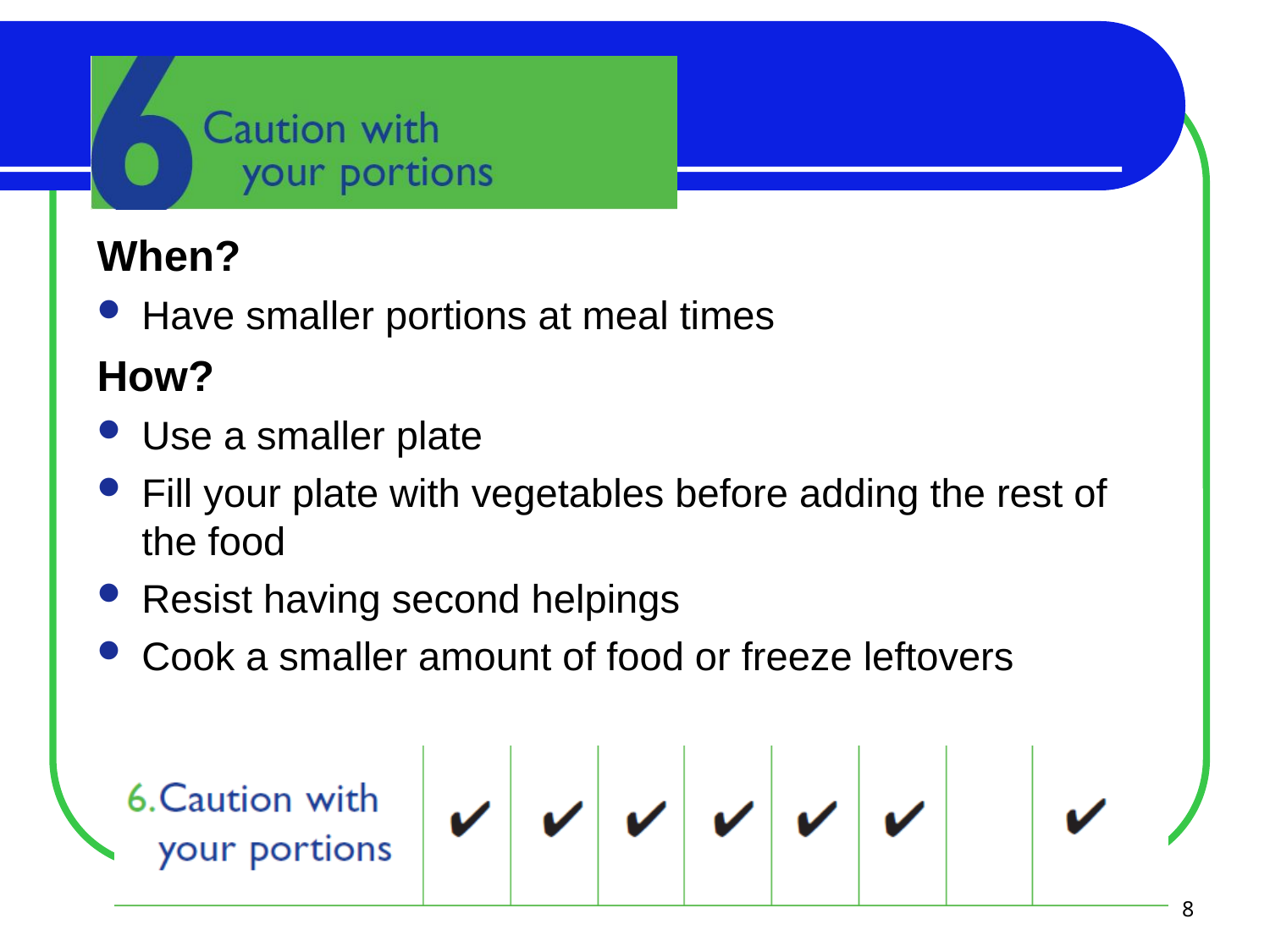

#
When?
Have smaller portions at meal times
How?
Use a smaller plate
Fill your plate with vegetables before adding the rest of the food
Resist having second helpings
Cook a smaller amount of food or freeze leftovers
<number>

## Slide 9
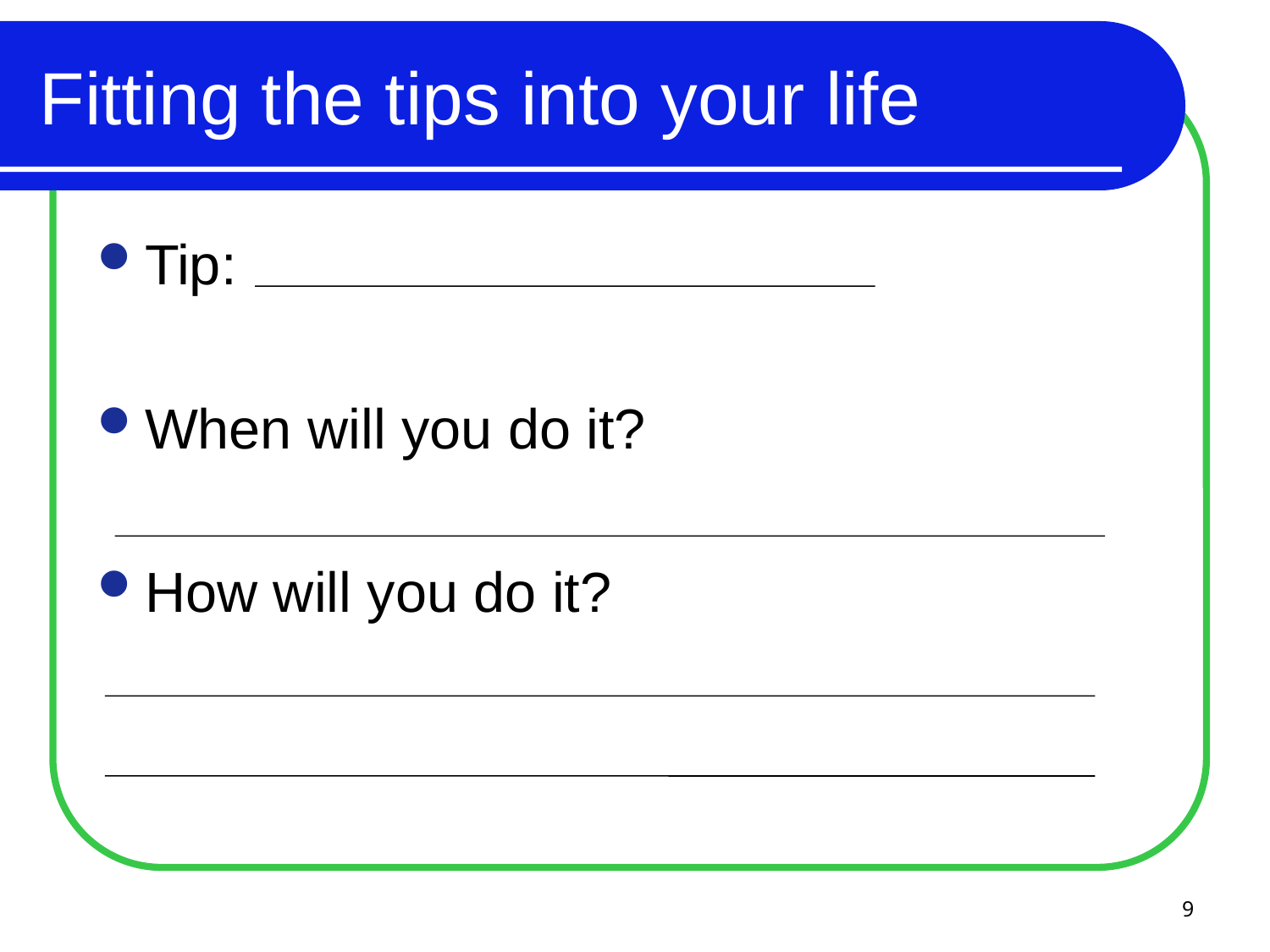

# Fitting the tips into your life
Tip:
When will you do it?
How will you do it?
<number>

## Slide 10
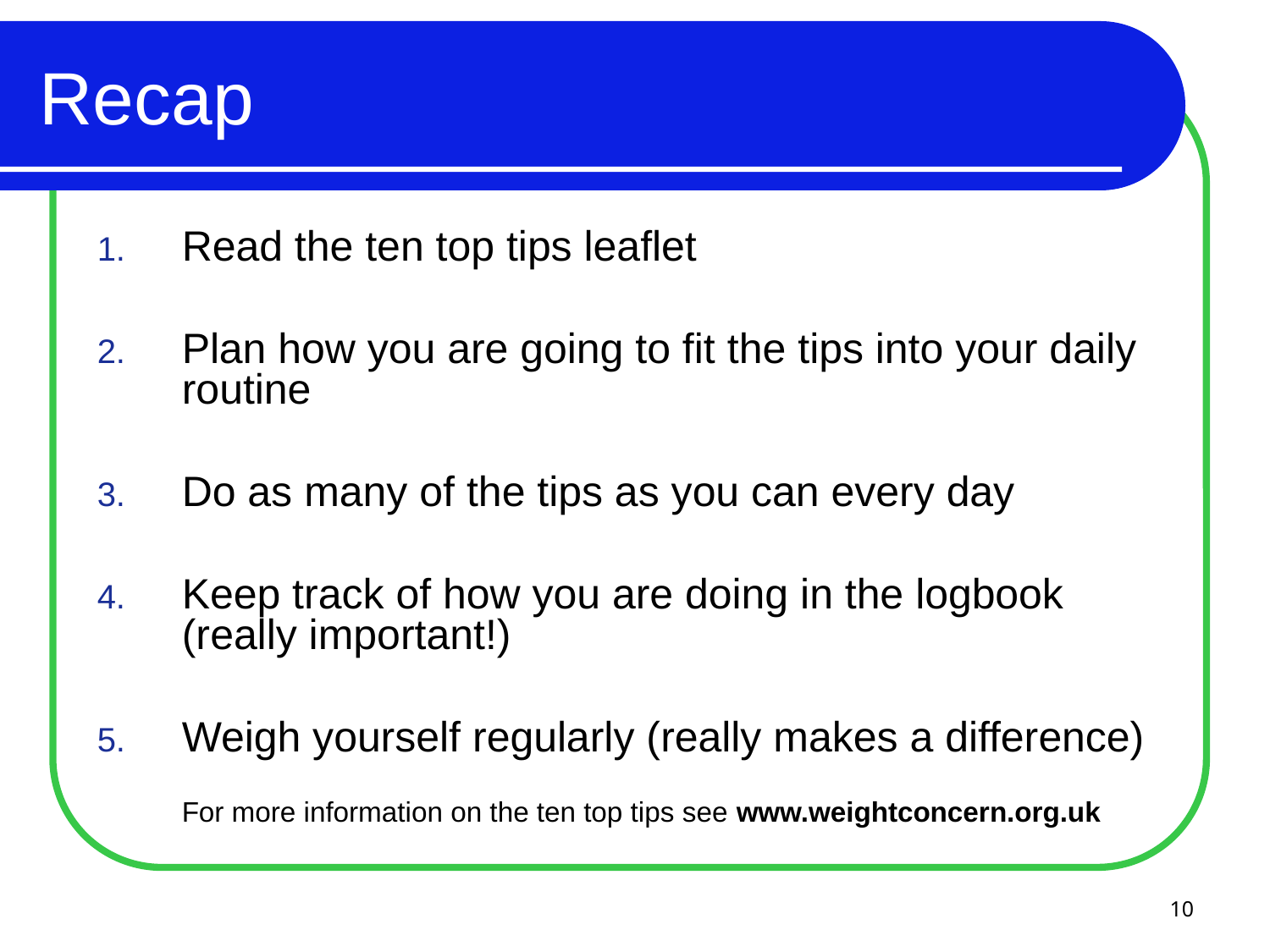

# Recap
Read the ten top tips leaflet
Plan how you are going to fit the tips into your daily routine
Do as many of the tips as you can every day
Keep track of how you are doing in the logbook (really important!)
Weigh yourself regularly (really makes a difference)
For more information on the ten top tips see www.weightconcern.org.uk
<number>

## Slide 11
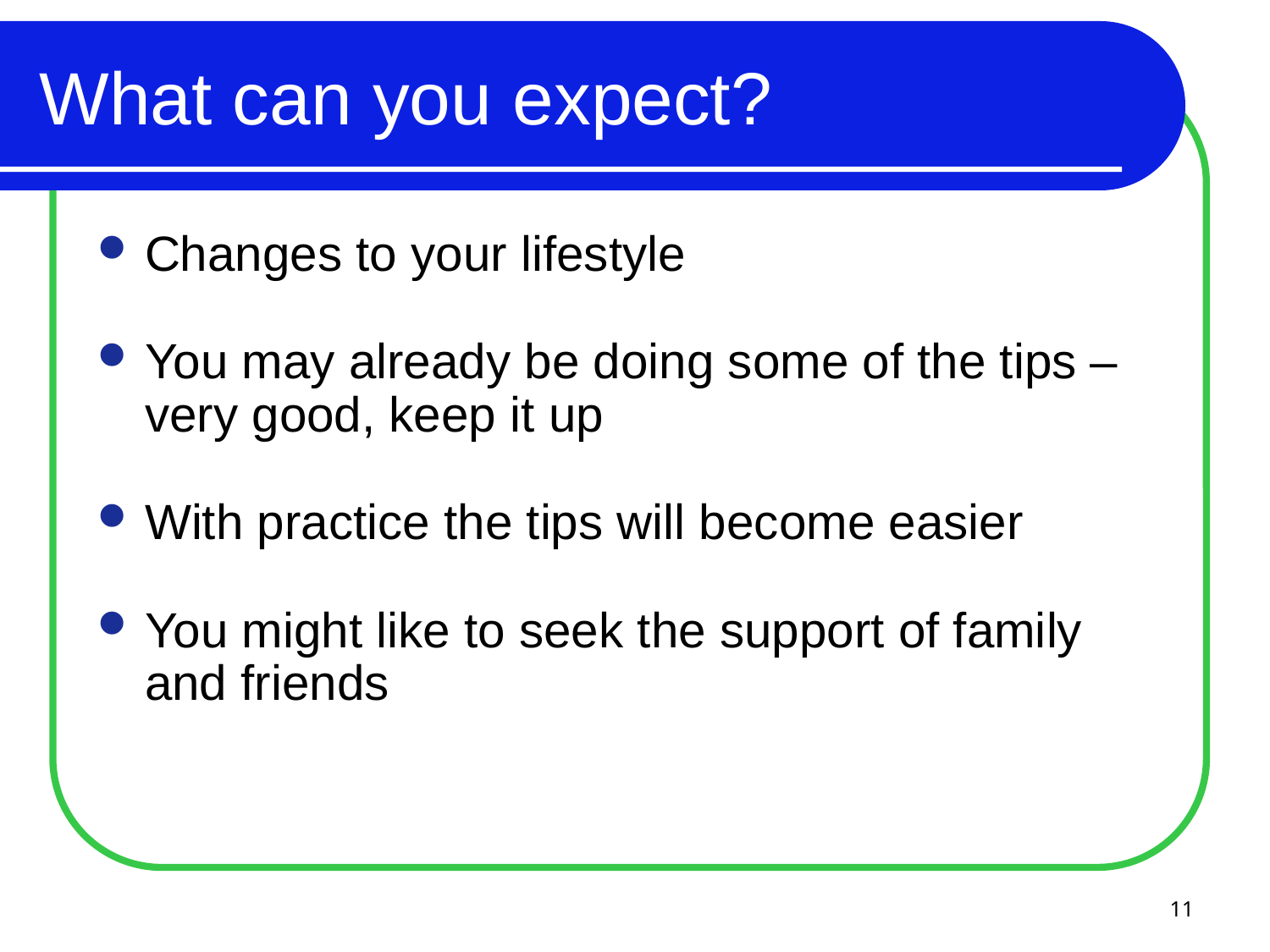

# What can you expect?
Changes to your lifestyle
You may already be doing some of the tips – very good, keep it up
With practice the tips will become easier
You might like to seek the support of family and friends
<number>

## Slide 12
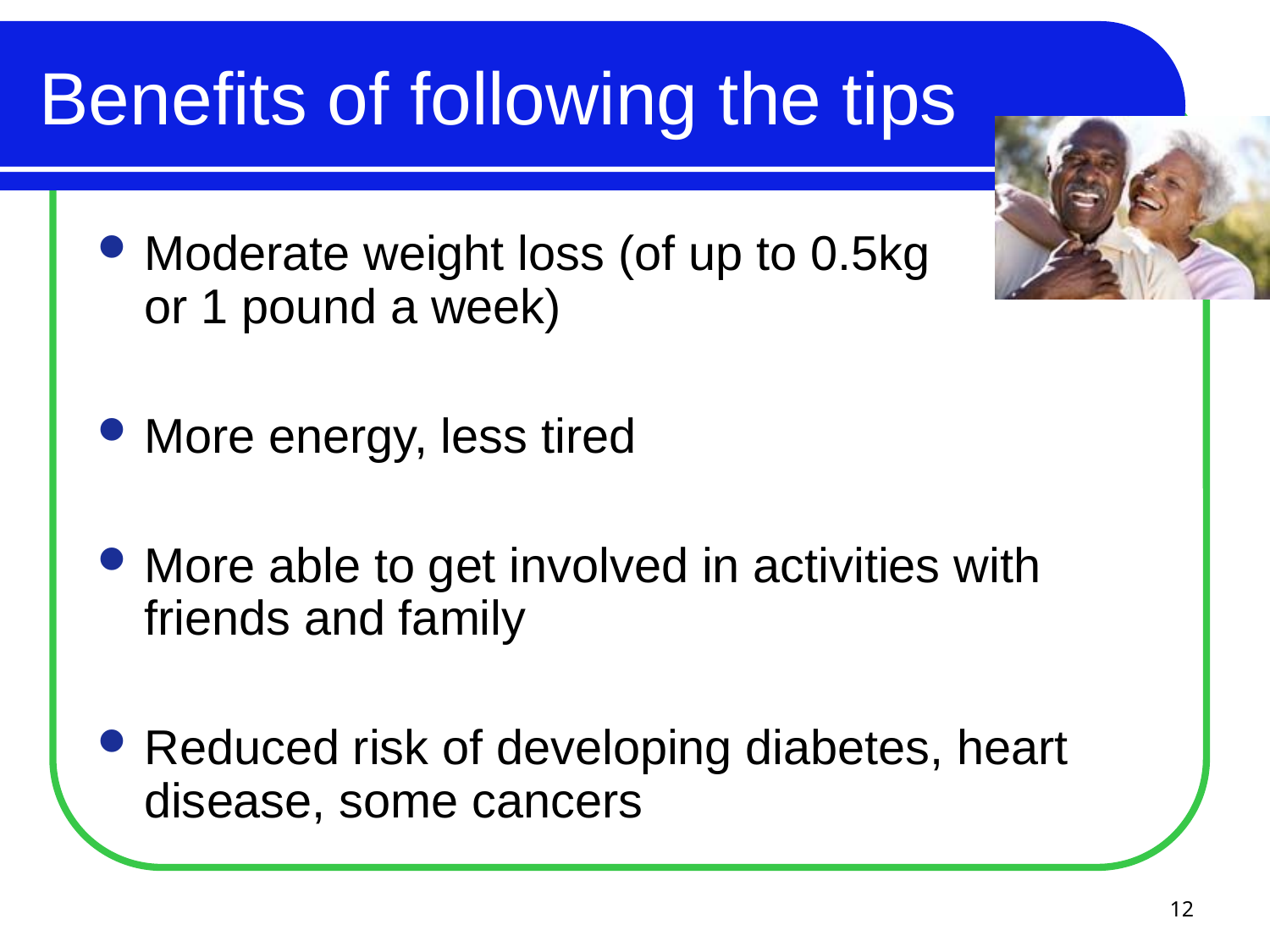

# Benefits of following the tips
Moderate weight loss (of up to 0.5kg or 1 pound a week)
More energy, less tired
More able to get involved in activities with friends and family
Reduced risk of developing diabetes, heart disease, some cancers
<number>

## Slide 13
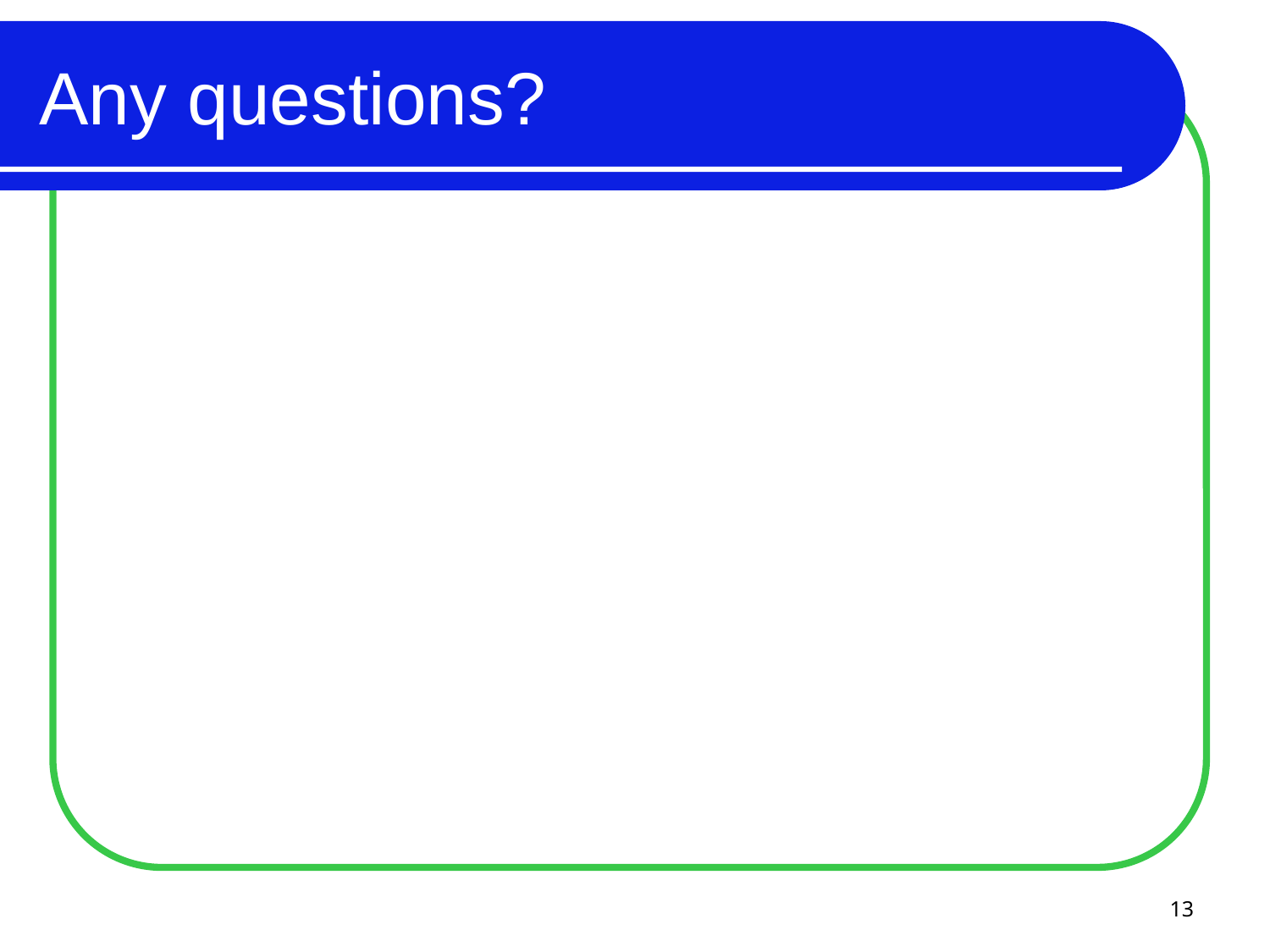

# Any questions?
<number>
