# Supplementary material for: A brief intervention for weight control based on habit-formation theory delivered through primary care: results from a randomised controlled trial
Source: Int J Obes (Lond). 2016 Nov 21;41(2):246–54. doi: 10.1038/ijo.2016.206 (PMC5300101; doi:10.1038/ijo.2016.206)
Supplement: Supplementary file 5 — Supplementary Information (DOCX 31 kb) [file 41366_2017_BFijo2016206_MOESM11_ESM.docx]

**Supplementary material: Difference between arms in weight change at 3-months, assuming Not at Random missing data pattern (MNAR)**

|  |  | δ_i_ = hypothetical difference in weight change between drop out and completers and non-completers in the intervention group (kg) | | | |
| --- | --- | --- | --- | --- | --- |
|  |  | **0** | **1.02** | **2.03** | **3.05** |
| δ_c_ = hypothetical difference in weight change between completers and non-completers in the control group (kg) | **0** | -0.88  [-1.50 to -0.27] | -0.58  [-1.19 to 0.03] | -0.28  [-0.9 to 0.34] | 0.02  [-0.61 to 0.66] |
|  | **1.02** | -1.17  [-1.78 to -0.55] | -0.86  [-1.48 to -0.25] | -0.56  [-1.19 to 0.06] | -0.26  [-0.89 to 0.38] |
|  | **2.03** | -1.45  [-2.07 to -0.82] | -1.14  [-1.77 to -0.52] | -0.84  [-1.47 to -0.21] | -0.54  [-1.18 to 0.10] |
|  | **3.05** | -1.73  [-2.36 to -1.1] | -1.43  [-2.06 to -0.79] | -1.13  [-1.77 to -0.48] | -0.82  [-1.47 to -0.17] |

[Type a quote from the document or the summary of an interesting point. You can position the text box anywhere in the document. Use the Text Box Tools tab to change the formatting of the pull quote text box.]

Plausibility of scenario:

| Likely |
| --- |
|  |
|  |
|  |
|  |
| Very unlikely |

MNAR pattern indicates that participants who dropped out from the study differ from the ones who completed the three-month follow-up, even after taking into account of the baseline characteristics. This analysis explores how the trial primary result would differ if this was to be the case, under a variety of scenarios.

δ_c_ and δ_i_ are the pattern mixture parameters, corresponding to different hypothetical values of how much less weight participants who dropped out from the study (i.e. outcome is missing) may have lost compared to those who did complete the three-months follow-up (i.e. outcome is observed).

The result reported in each cell is the estimated difference between arms in primary outcome (and 95% confidence intervals) for the corresponding δ_c_ and δ_i_, after adjustment for baseline weight and general practice.

For example, the bottom right-hand corner cell reads: “Assuming participants who dropped out from the study have lost on average 3.05kg less than those who completed the follow-up, both in control and intervention arm, the estimated difference between arms in weight loss at 3 months would be of 0.82kg”.

Colours corresponds to likeliness of each scenario, under the assumptions that i) δ_c_ and δ_i_ are likely to be around +0.5 standard deviation of the observed weight change (participants who completed the study are more likely to have lose slightly more weight than those who did not complete it), and ii) δ_c_ and δ_i_ are likely to be similar to each other (it is unlikely that the pattern of missingness is very different between the 2 arms).

The range of δ_c_ and δ_i_ was decided a priori, as a function of the standard deviation of the observed weight change. It was assumed 0 to 1 standard deviation should be sufficient to cover all reasonably possible MNAR scenarios.
